# Screening and functional verification of drought resistance-related genes in castor bean seeds

**DOI:** 10.1186/s12870-024-04997-7

**Published:** 2024-06-03

**Authors:** Zhiyan Wang, Rui Luo, Qi Wen, Xiaotian Liang, Huibo Zhao, Yong Zhao, Mingda Yin, Yanpeng Wen, Xuemei Hu, Fenglan Huang

**Affiliations:** 1 College of Life Science and Food Engineering, Inner Mongolia Minzu University, Tongliao, 028000 Inner Mongolia China; 2 Agronomy College, Jilin Agricultural University, Changchun, 130118 China; 3 National Oat Improvement Center, Baicheng Academy of Agricultural Sciences, Baicheng, 137000 China; 4 China National Rice Research Institute, Hangzhou, 311400 China; 5 School of Life Sciences, Laboratory of Genetics, Baicheng Normal University, Baicheng, 137000 China; 6 Key Laboratory of Castor Breeding of the National Ethnic Affairs Commission of the People’s Republic of China, Tongliao, 028000 Inner Mongolia China; 7 Inner Mongolia Key Laboratory of Castor Breeding and Comprehensive Utilization, Tongliao, 028000 Inner Mongolia China

**Keywords:** Castor bean, Drought stress, *RcECP63*, *RcDDX31*, *RcA/HD1*

## Abstract

Drought is one of the natural stresses that greatly impact plants. Castor bean (*Ricinus communis* L.) is an oil crop with high economic value. Drought is one of the factors limiting castor bean growth. The drought resistance mechanisms of castor bean have become a research focus. In this study, we used castor germinating embryos as experimental materials, and screened genes related to drought resistance through physiological measurements, proteomics and metabolomics joint analysis; castor drought-related genes were subjected to transient silencing expression analysis in castor leaves to validate their drought-resistant functions, and heterologous overexpression and backward complementary expression in Arabidopsis thaliana, and analysed the mechanism of the genes' response to the participation of Arabidopsis thaliana in drought-resistance.

Three drought tolerance-related genes, *RcECP 63*, *RcDDX 31* and *RcA/HD1*, were obtained by screening and analysis, and transient silencing of expression in castor leaves further verified that these three genes corresponded to drought stress, and heterologous overexpression and back-complementary expression of the three genes in Arabidopsis thaliana revealed that the function of these three genes in drought stress response.

In this study, three drought tolerance related genes, *RcECP 63*, *RcDDX 31* and *RcA/HD1*, were screened and analysed for gene function, which were found to be responsive to drought stress and to function in drought stress, laying the foundation for the study of drought tolerance mechanism in castor bean.

**Supplementary Information:**

The online version contains supplementary material available at 10.1186/s12870-024-04997-7.

## Introduction

Plant growth and development are mainly affected by many abiotic stresses such as salinity stress, drought stress, cold stress, etc., of which the impact of drought stress on plant growth is particularly prominent, in the process of plant growth and development is vulnerable to salinity, wet flooding, high temperature, low temperature, heavy metal ions and drought and other abiotic environmental stresses, which will reduce the yield of the crop, which drought caused by the most reduction in crop yields [1–3], therefore, the study of the drought tolerance of the plant and the cultivation of drought-resistant plant varieties in order to cope with the environment is more necessary.

Plant response to abiotic stresses such as drought is achieved through the regulation of gene expression. Plant responses to drought stress signals include both physiological responses at the cellular level and changes in gene expression. At the cellular level, the recognition, transduction and response to intercellular messengers through receptor cells will ultimately regulate the expression and metabolism of specific genes to improve drought resistance in plants [4, 5]. In terms of gene expression, drought-resistant gene expression is altered by signal transduction after drought stress in plants thereby increasing drought resistance.

Castor bean (*Ricinus communis* L.) is an annual or perennial herbaceous plant species belonging to the genus *Ricinus* in the family Euphorbiaceae [6, 7]. It is an oil crop with high economic and use value [8]. Castor oil has many application values, and it is hailed as a renewable “petroleum resource” as the only vegetable oil that can replace petroleum. Therefore, it is of great significance to study castor bean.

In this study, we screened out the genes involved in drought stress regulation in castor bean embryos at the germination stage by using differential proteomics, comparative metabolomics, and reverse transcription–quantitative PCR (RT-qPCR) analysis. We overexpressed and complementarily expressed these genes into *Arabidopsis thaliana* and verified the functions of these genes through the analysis of gene expression levels, measurement of relevant physiological indicators, and phenotype analysis of T3 generation homozygous *A. thaliana*, aiming to lay a theoretical basis for determining the drought resistance mechanisms of castor bean.

## Results and analysis

### Physiological studies on the response of castor embryos to drought stress during germination

#### Measurement of Catalase (CAT) activity

**Fig. 1:**
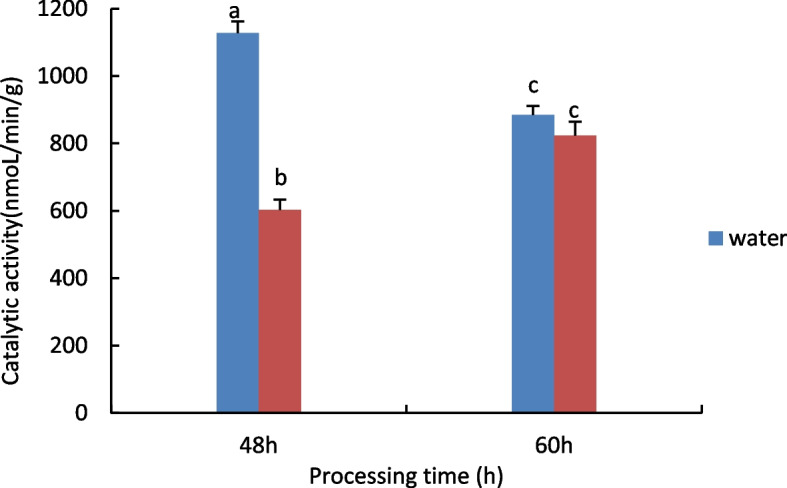
Results of CAT activity assay of castor embryos with different treatments. Note: Different lower case letters represent significant differences (*p* < 0.05)

The results are shown in Fig. 1, with increasing processing time, water treatment shows a downward trend, 15% PEG treatment showed an increasing trend.

#### Measurement of Superoxide dismutase (SOD) activity

**Fig. 2:**
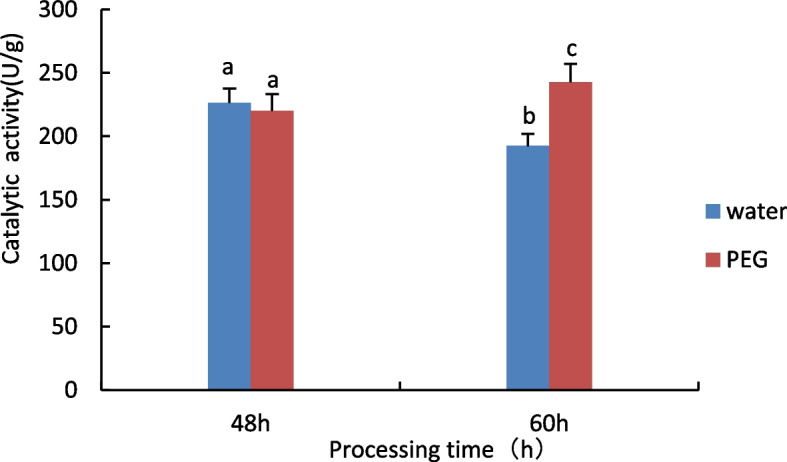
Results of SOD activity assay of castor embryos with different treatments. Note: Different lower case letters represent significant differences (*p* < 0.05)

The results are shown in Fig. 2, no significant difference in SOD activity between the 48 h water treatment and the 15% PEG 6000 treatment, when treated with stress for 60 h, compared to control, the SOD activity of castor seed embryos showed an increasing trend under 15% PEG treatment.

#### Measurement of Peroxidase (POD) activity

**Fig. 3:**
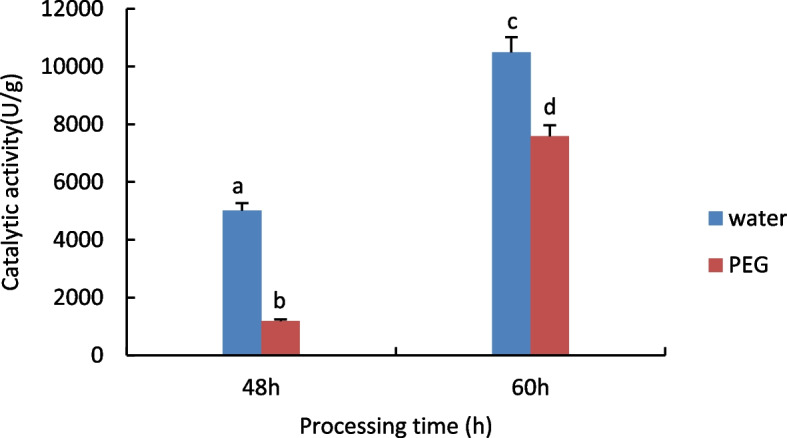
Results of POD activity assay of castor embryos with different treatments. Note: Different lower case letters represent significant differences (*p* < 0.05)

The results are shown in Fig. 3, with increasing treatment time POD activity increased significantly in both the treated and control groups, and in the comparison of different treatments at the same time, the activity of POD in the control group was significantly higher than that in the treated group.

#### Measurement of Glutathione S-transferase (GST) activity

**Fig. 4:**
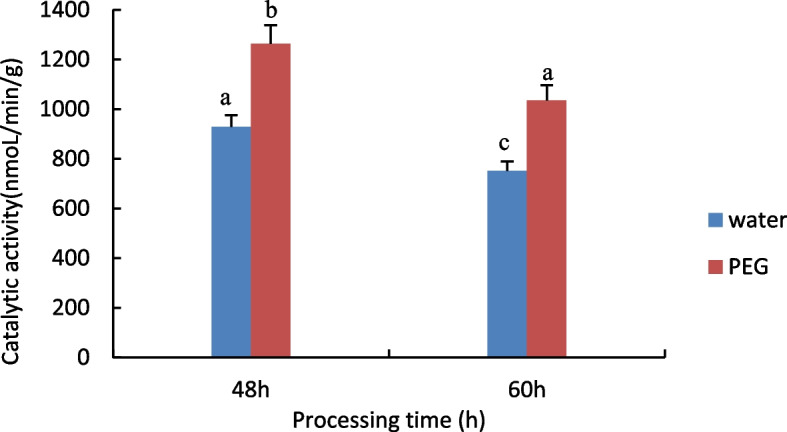
Results of GST activity assay of castor embryos with different treatments. Note: Different lower case letters represent significant differences (*p* < 0.05)

The results are shown in Fig. 4, with increasing processing time, GST activity was significantly reduced in both the control and treated groups, the treated group had higher GST activity than the control group.

#### Measurement of Total antioxidant capacity (T-AOC) capacity

**Fig. 5:**
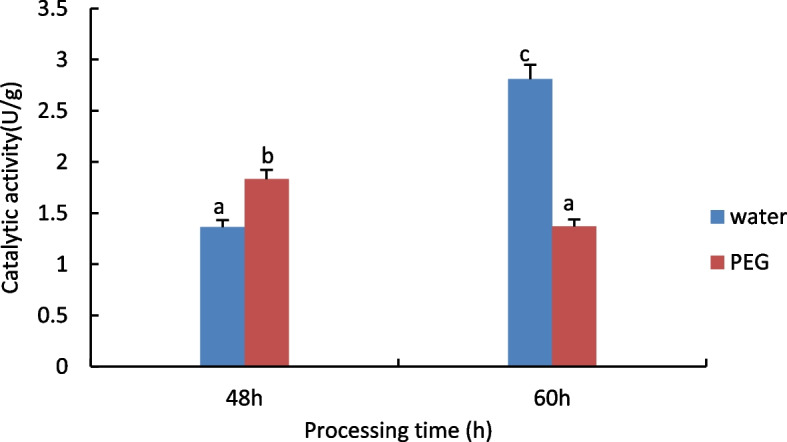
Results of T-AOC activity assay of castor embryos with different treatments. Note: Different lower case letters represent significant differences (*p* < 0.05)

The results are shown in Fig. 5, compared to control, with increasing processing time, significant decrease in T-AOC activity of seed embryos, significant increase in T-AOC activity in the control group.

#### Measurement of Malondialdehyde (MDA) activity

**Fig. 6:**
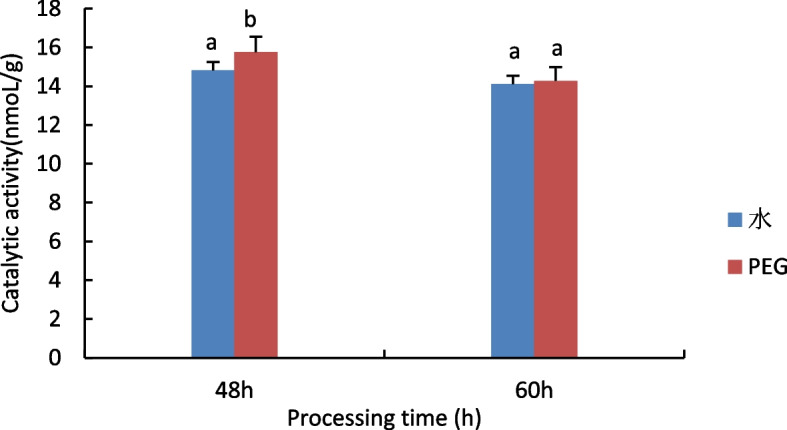
Results of MDA activity assay of castor embryos with different treatments. Note: Different lower case letters represent significant differences (*p* < 0.05)

The results are shown in Fig. 6, compared to control, with increasing processing time, increase in MDA levels, while with increasing stress time, MDA content decreased in both water and 15% PEG 6000 treatments.

#### Measurement of Hydrogen peroxide (H2O2) activity

**Fig. 7:**
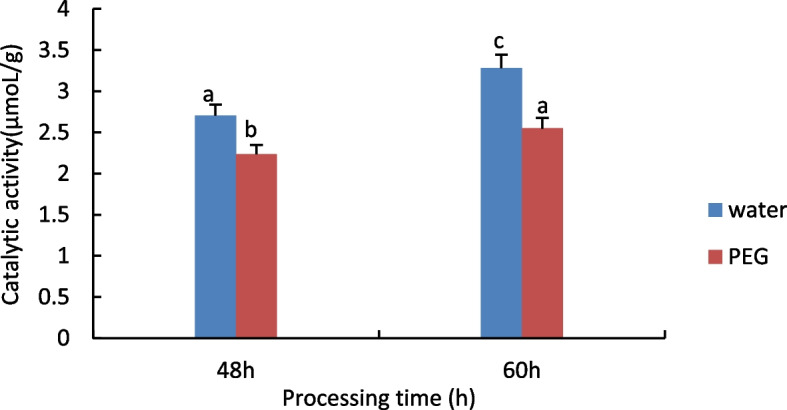
Results of H_2_O_2_ activity assay of castor embryos with different treatments. Note: Different lower case letters represent significant differences (*p* < 0.05)

The results are shown in Fig. 7, the level of hydrogen peroxide in both the treatment and control groups increased significantly with time, compared to the control group, The water treatment was significantly higher than the PEG treatment in the amount of hydrogen peroxide for the same treatment time.

#### Measurement of Proline (Pro) activity

**Fig. 8:**
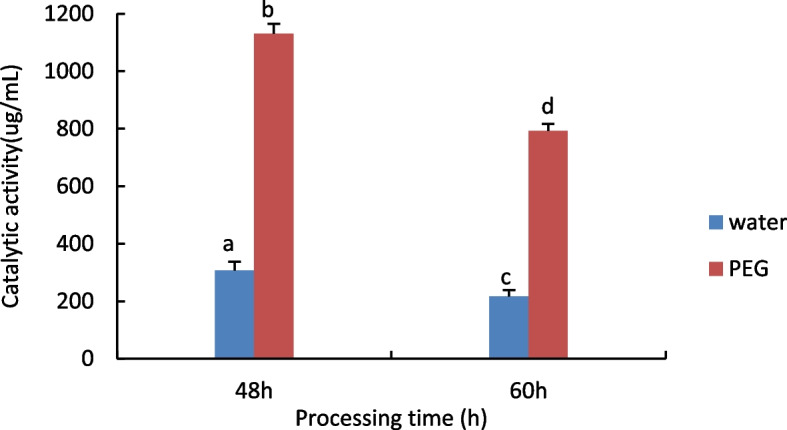
Results of Pro activity assay of castor embryos with different treatments. Note: Different lower case letters represent significant differences (*p* < 0.05)

The results are shown in Fig. 8, compared to control, Pro content in embryos of all samples under PEG stress increased, Pro content was reduced in both water and PEG-treated castor embryos.The accumulation of Pro plays a decisive role in the resistance of the plant body, and Pro prevents changes in plasma membrane permeability and has a protective effect on the integrity of the plasma membrane, and the results of this experiment suggest that Pro responds to drought stress by exerting an osmoregulatory effect.

#### Determination of hydroxyl radical scavenging capacity

**Fig. 9:**
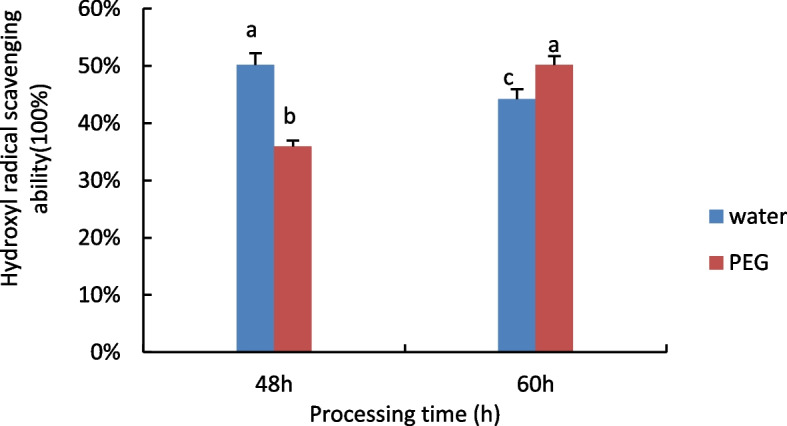
Results of Pro activity assay of castor embryos with different treatments. Note: Different lower case letters represent significant differences (*p* < 0.05)

The results are shown in Fig. 9, hydroxyl radical scavenging capacity decreases with time under water treatment conditions, while rising under 15% PEG treatment.

The transient silencing expression analysis of castor leaves revealed that three genes, *RcECP 63*, *RcDDX 31* and *RcA/HD1*, were silenced, resulting in more severe surface wilting of castor leaves under drought stress conditions compared with the control; the expression of the three genes was significantly down-regulated after silencing; a significant increase in MDA content was found in the determination of physiological indicators, while the content of Pro, hydroxyl radical scavenging capacity and T-AOC showed a decreasing trend, indicating that the three selected genes have functions in the response to leaf involvement in drought stress, and it is hypothesized that these three genes are also involved in drought stress during the sprouting stage of castor.

### Differential proteomic and widely targeted metabolomic analyses of castor bean embryos at the germination stage

#### Differential proteomics

##### Differentially expressed protein analysis

**Fig. 10:**
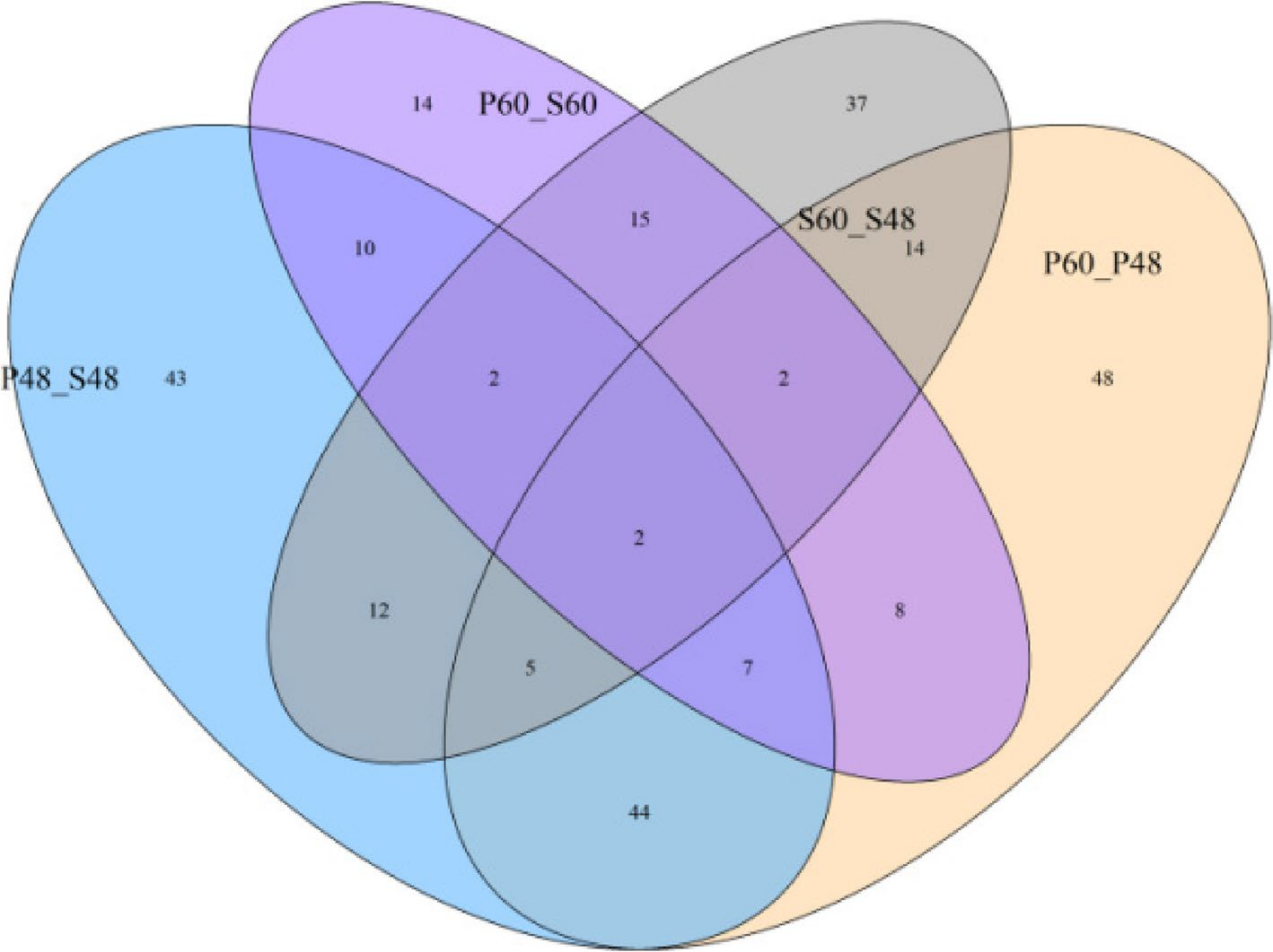
Venn diagram of differentially expressed proteins

**Fig. 11:**
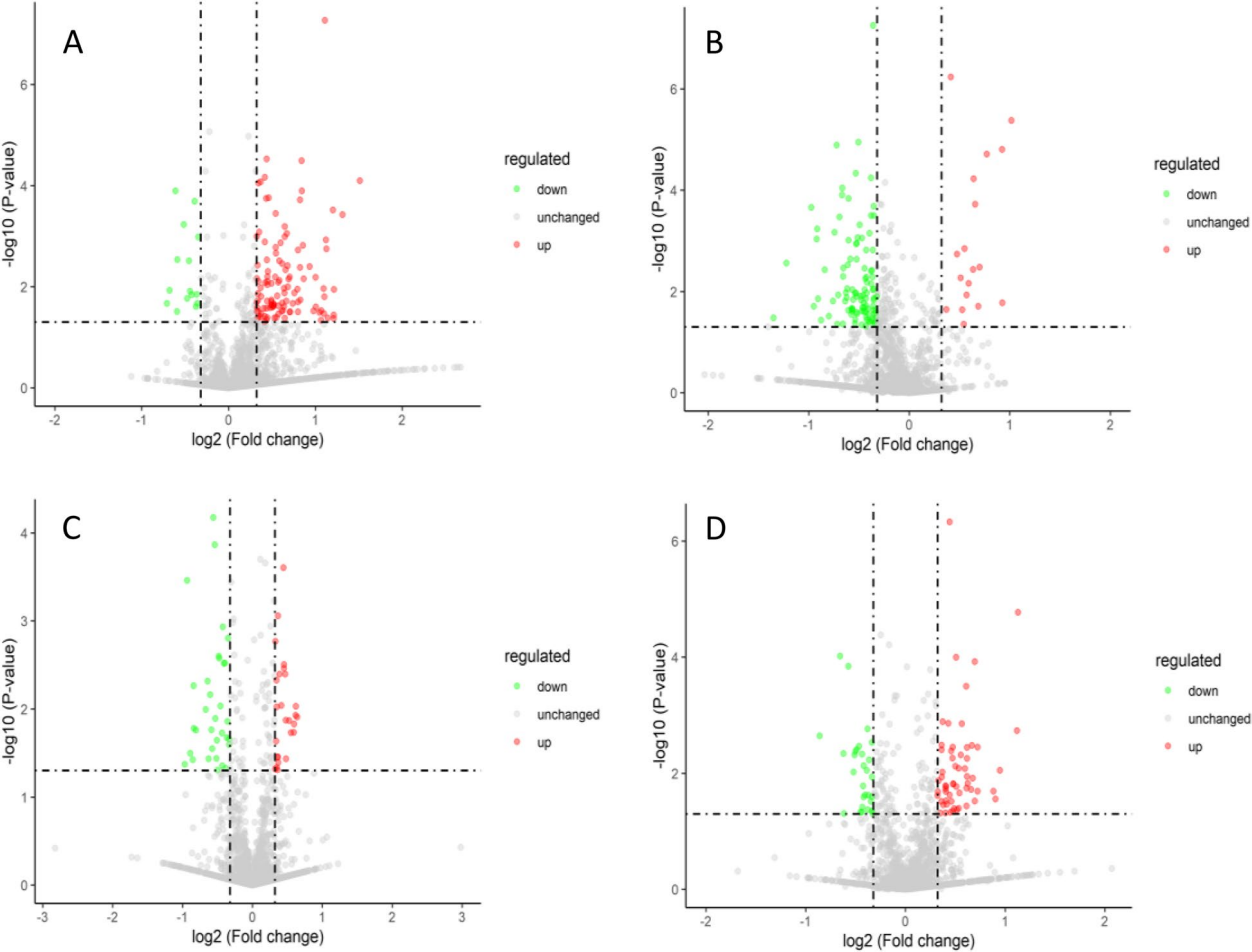
Volcano plots of differentially expressed proteins. Note: A-D show the differentially expressed proteins screened out in the four comparison groups: P48_VS_P60, S48_VS_P48, S60_VS_P60, and S48_VS_S60, respectively. The green dots represent the differentially expressed proteins with downregulated expression, the red dots represent the differentially expressed proteins with upregulated expression, and the gray dots represent the detectable proteins with no significant changes in expression

The iTRAQ technique was used to perform proteomic analysis on castor embryo samples with different treatments. The total number of identified peptides was 13,971; the number of identified unique peptides was 9,592; and the number of identified proteins was 2,101. The Venn diagram and volcano plots of differentially expressed proteins are shown in Figs. 10 and 11. In the comparison group P48_VS_P60, there were 115 and 15 upregulated and downregulated proteins, respectively. In the comparison group S48_VS_P48, there were 18 and 106 upregulated and downregulated proteins, respectively. In the comparison group S60_VS_P60, there were 26 and 33 upregulated and downregulated proteins, respectively. In the comparison group S48_VS_S60, there were 59 and 29 upregulated and downregulated proteins, respectively.

##### Hierarchical clustering results of differentially expressed proteins

**Fig. 12:**
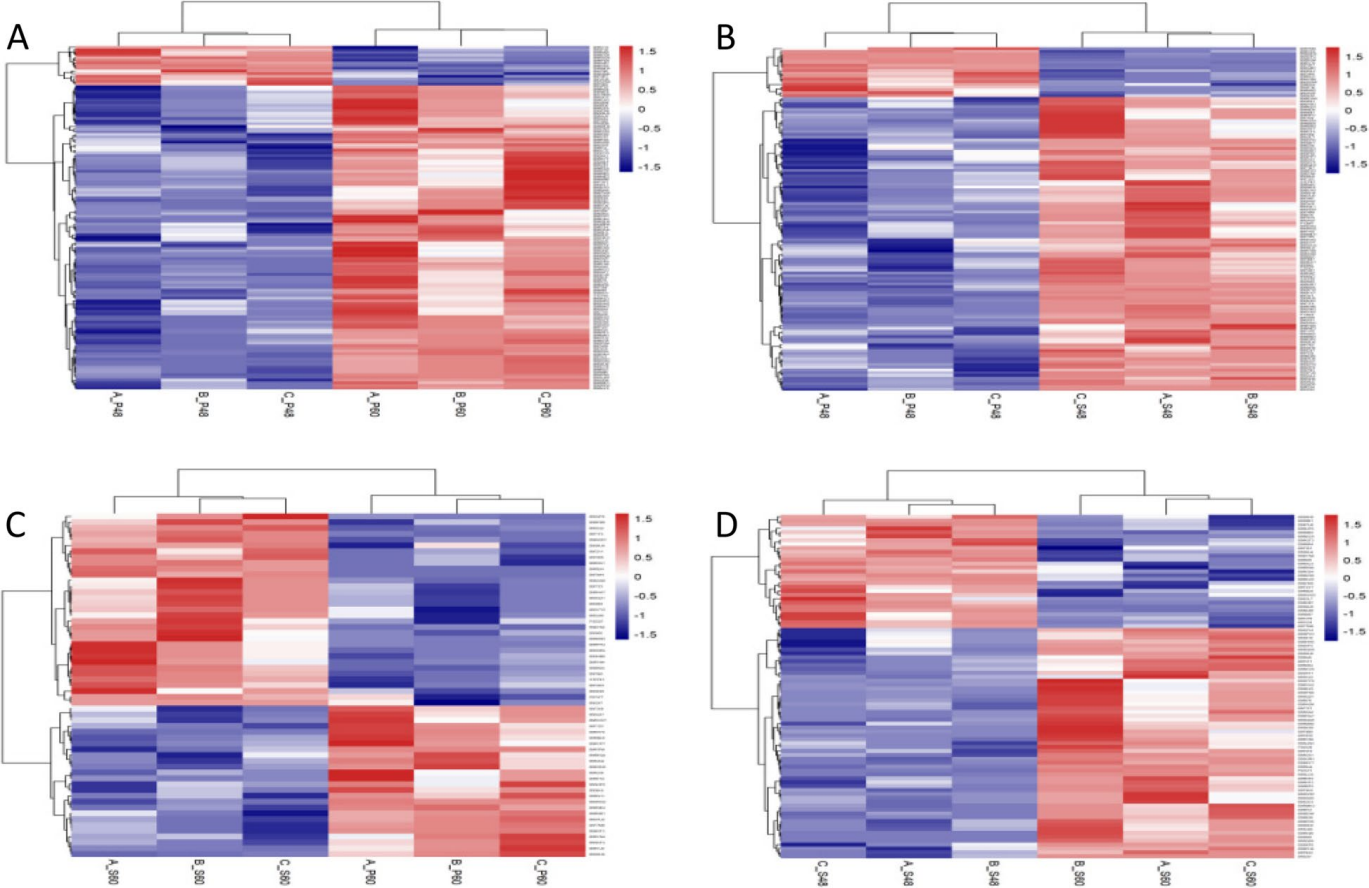
Hierarchical clustering analysis of differentially expressed proteins. Note: A-D show the results of hierarchical clustering analysis of the differentially expressed proteins in the four comparison groups P48_VS_P60, S48_VS_P48, S60_VS_P60, and S48_VS_S60, respectively

Figure 12 shows the results of hierarchical clustering analysis of the selected differentially expressed proteins. As the treatment concentration and treatment duration increased, the number of upregulated proteins significantly increased. We speculate that the longer drought stress elicited a more sensitive response of castor bean embryos to the external environment.

##### Gene Ontology functional annotation analysis of differentially expressed proteins

**Fig. 13:**
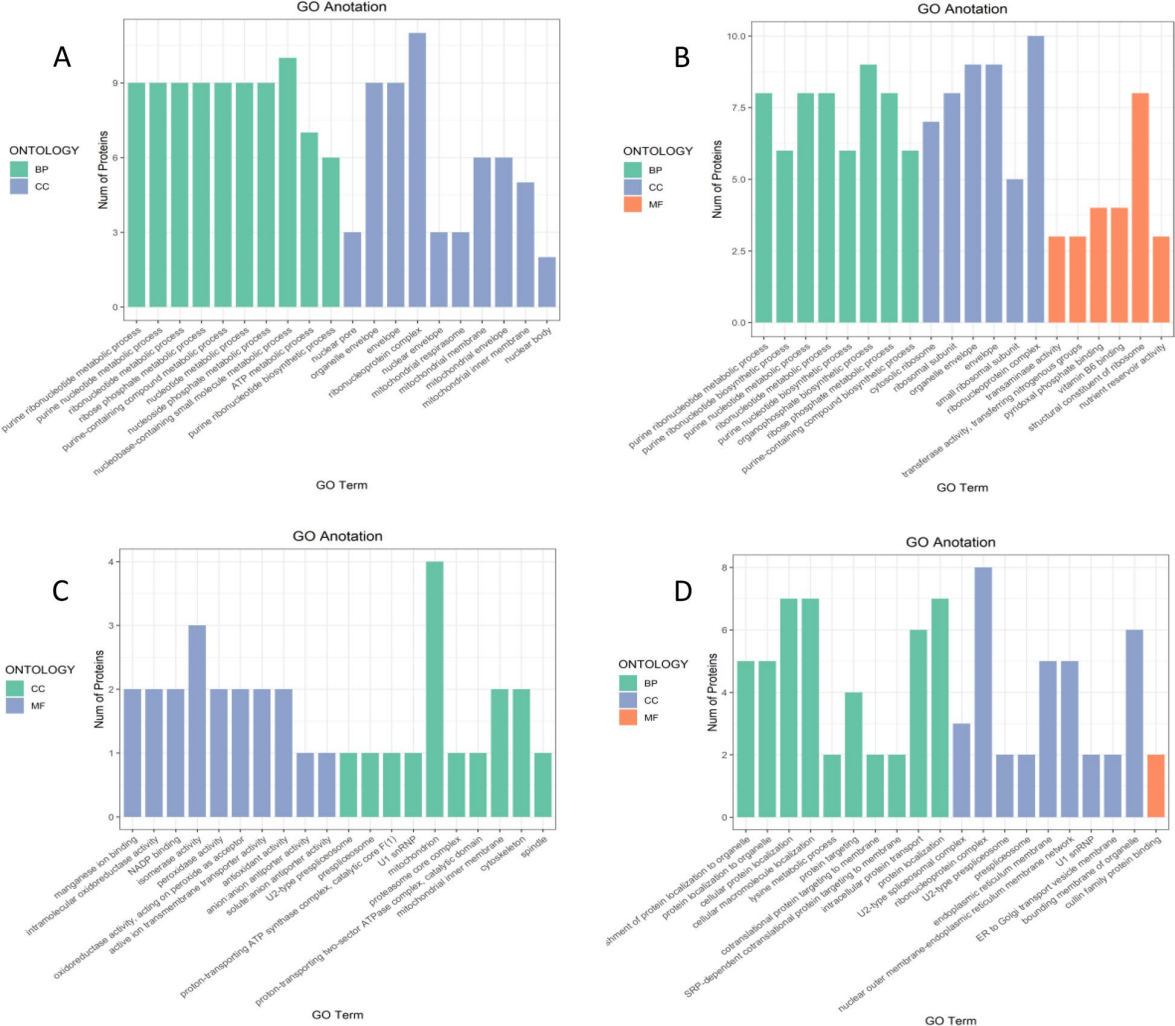
Gene Ontology functional annotation analysis of differentially expressed proteins. Note: A-D show the annotation results of differentially expressed proteins in the four comparison groups P48_VS_P60, S48_VS_P48, S60_VS_P60, and S48_VS_S60, respectively

To further study the biological processes involving differentially expressed proteins, Gene Ontology functional enrichment analysis was performed on the differentially expressed proteins. The results in Fig. 13 indicate that the differentially expressed proteins participated in the whole life cycle through biological processes, cellular components, and molecular functions. As the drought stress is prolonged, the external environment influences the germination of castor bean seeds by changing not only the material and energy transport but also the biosynthesis and metabolism of substances.

#### Widely targeted metabolomics

##### Volcano plots of differentially expressed metabolites

**Fig. 14:**
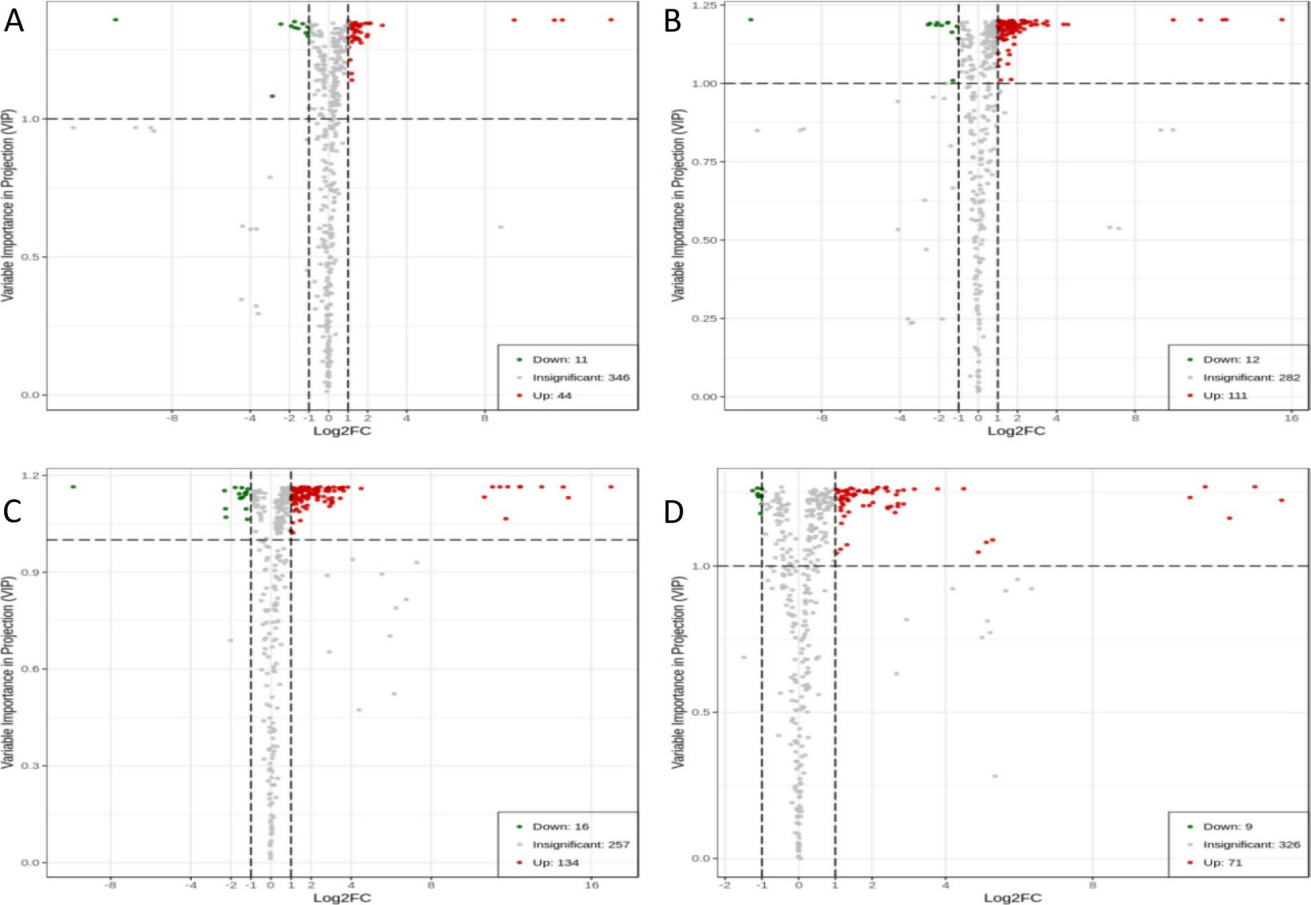
Volcano plots of differentially expressed metabolites. Note: A-D show the volcano plots of the differentially expressed metabolites in the four comparison groups P48_VS_P60, S48_VS_P48, S60_VS_P60, and S48_VS_S60, respectively. VIP represents the significance of the difference in a given metabolite between groups. Log_2_FC represents the fold change of the differentially expressed metabolite. The green dots represent the downregulated metabolites, the red dots represent the upregulated metabolites, and the gray dots represent the detectable metabolites with no significant changes in concentration

To analyze the fold changes and intergroup differences in the metabolite levels in the samples, the differentially expressed metabolites of the four groups (P48_VS_P60, S48_VS_P48, S60_VS_P60, and S48_VS_S60) of samples were analyzed by volcano plots. The results are shown in Fig. 14. The variable importance in projection (VIP) values of all metabolites were greater than 1, which indicates that the differential expression of metabolites was significant and that the screening of differentially expressed metabolites was reliable. Moreover, as the PEG 6000 concentration and the stress duration increased, the differentially expressed metabolites of the castor bean embryos changed more obviously, which indicates that abiotic stress had a more significant regulatory effect on the metabolites.

##### Differentially expressed metabolite screening results

**Fig. 15:**
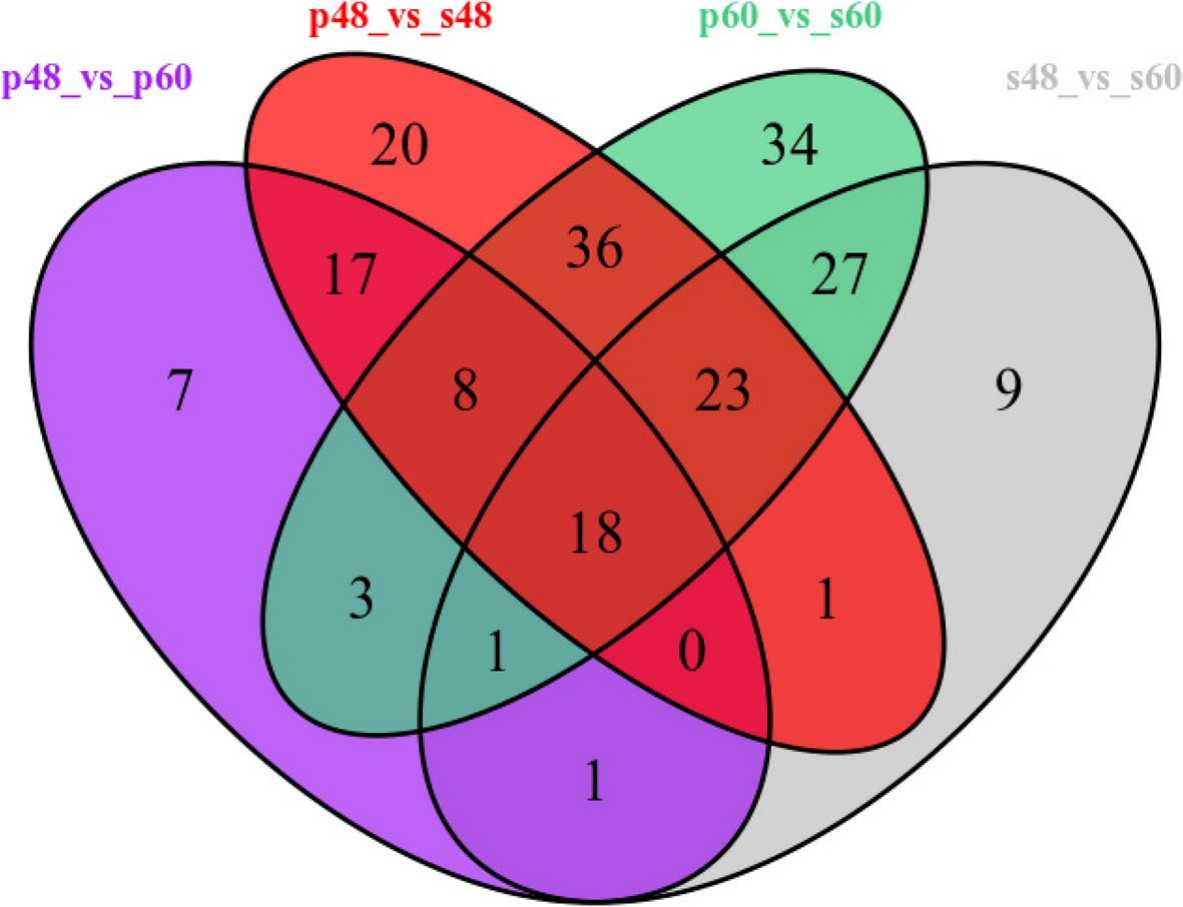
Venn diagram of differentially expressed metabolites in groups P48_VS_P60, S48_VS_P48, S60_VS_P60, and S48_VS_S60

A total of 408 differentially expressed metabolites were screened out in the four groups (P48_VS_P60, S48_VS_P48, S60_VS_P60, and S48_VS_S60) of samples. Among them, 123 differentially expressed metabolites were identified in P48_VS_S48, 55 were identified in P48_VS_P60, 80 were identified in S48_VS_S60, and 150 were identified in P60_VS_S60. The Venn diagram of differentially expressed metabolites in the four groups is shown in Fig. 15.

##### KEGG functional annotation analysis of differentially expressed metabolites

**Fig. 16:**
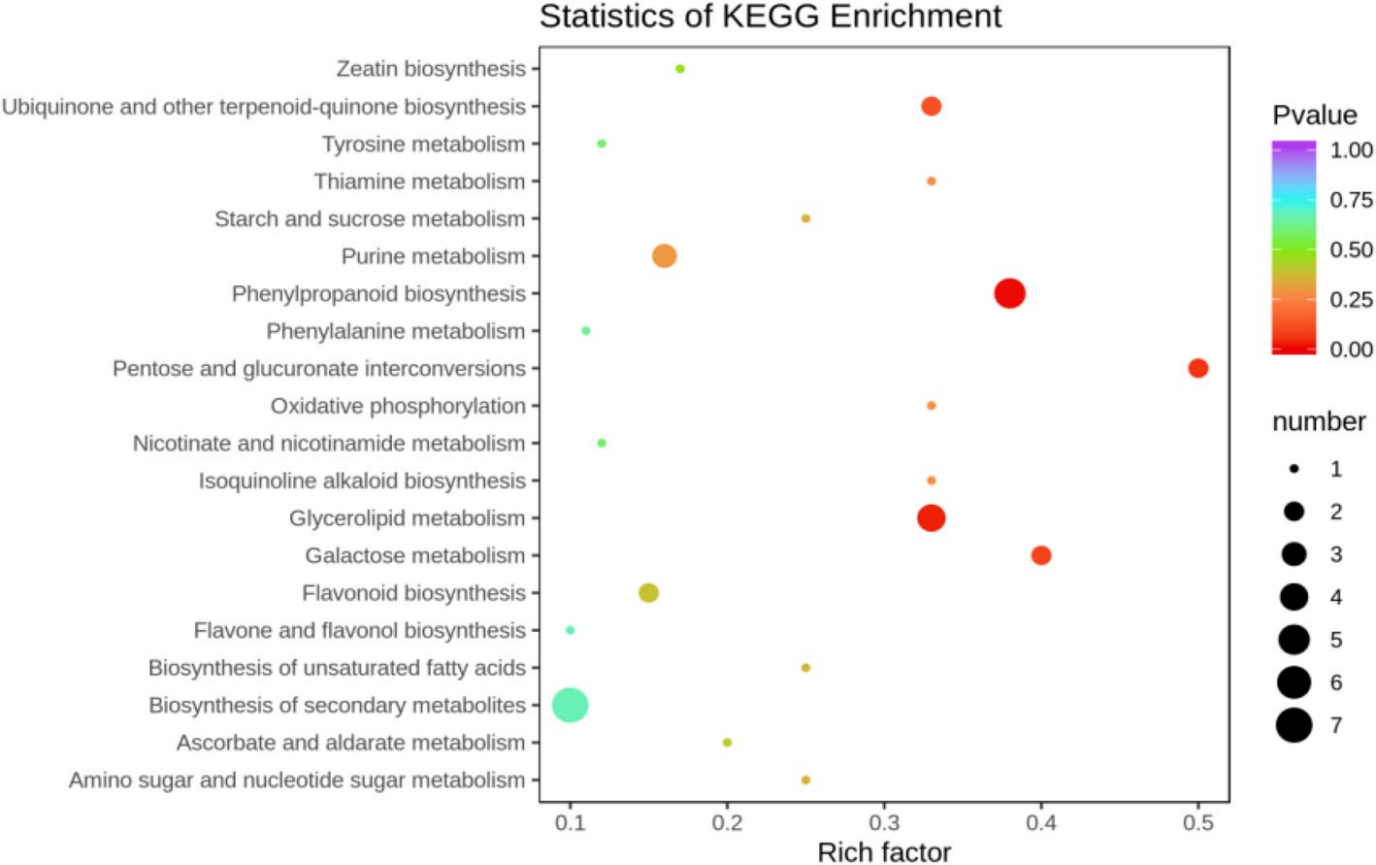
KEGG enrichment analysis of the differentially expressed metabolites in group P48_VS_P60

**Fig. 17:**
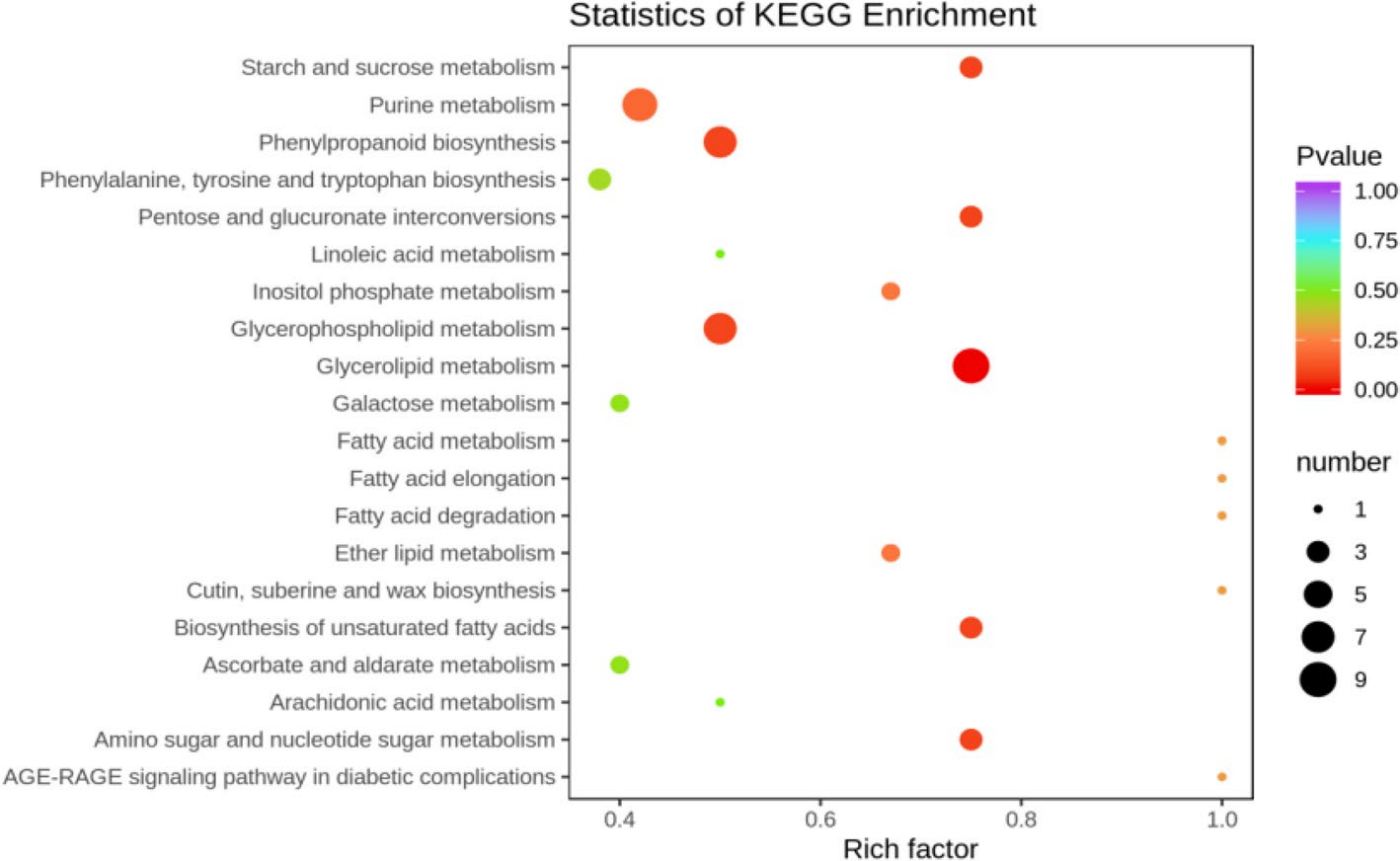
KEGG enrichment analysis of the differentially expressed metabolites in group P48_VS_S48

**Fig. 18:**
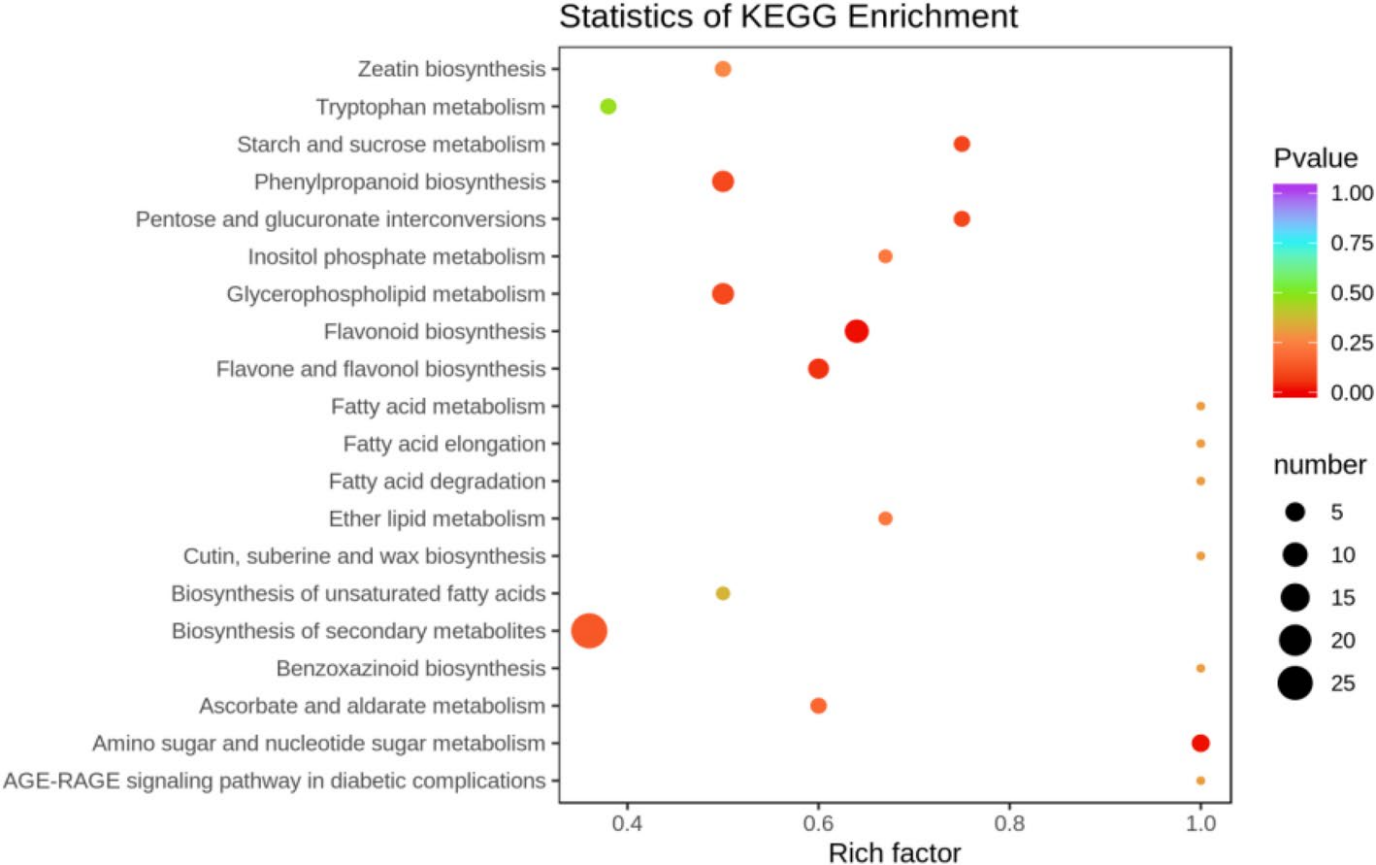
KEGG enrichment analysis of the differentially expressed metabolites in group P60_VS_S60

**Fig. 19:**
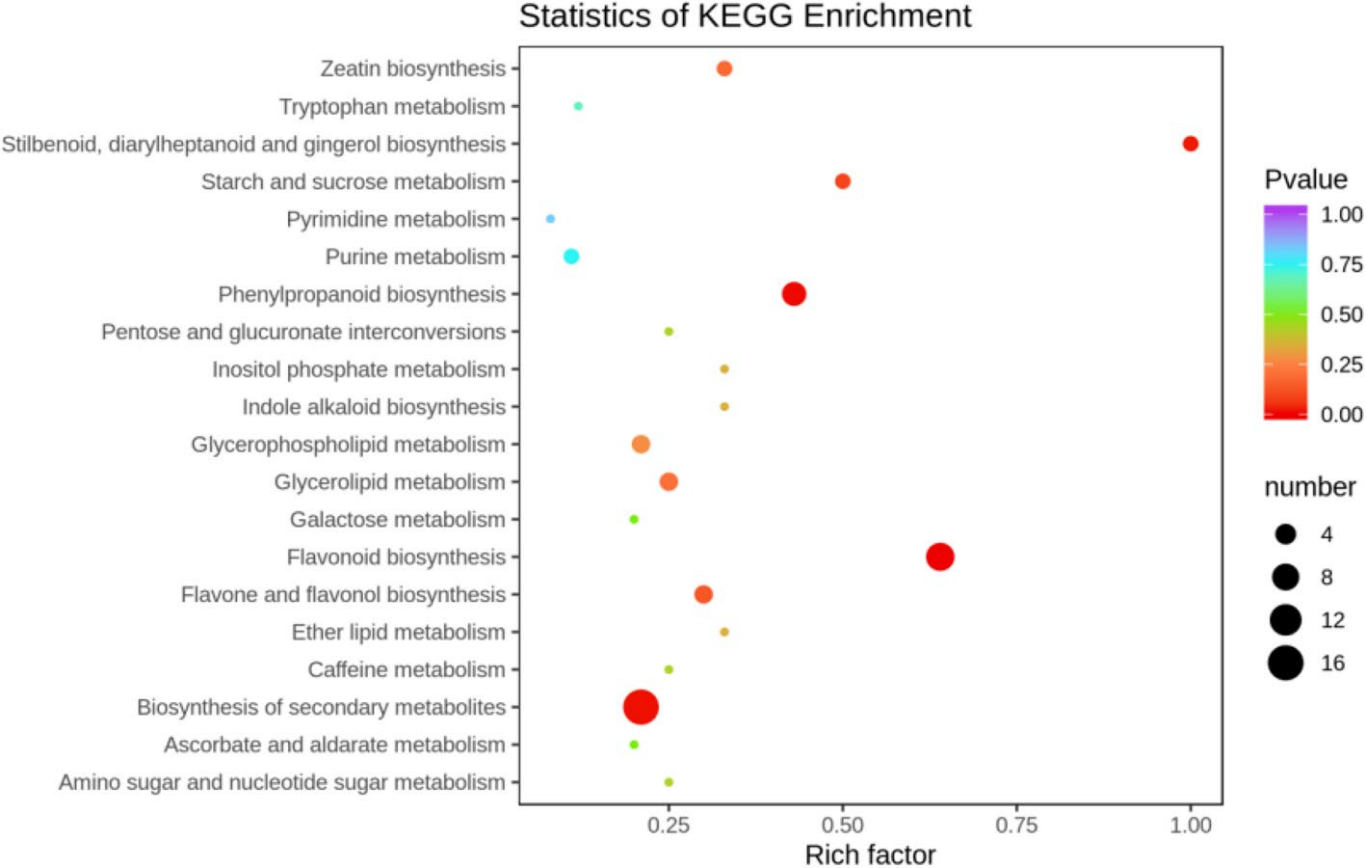
KEGG enrichment analysis of the differentially expressed metabolites in group S48_VS_S60

The differentially expressed metabolites screened out in the four comparison groups (P48_VS_P60, S48_VS_P48, S60_VS_P60, and S48_VS_S60) were subjected to functional annotation analysis of KEGG metabolic pathways. The results in Figs. 16, 17, 18 and 19 show that the highest proportion of differentially expressed metabolites in group P48_VS_P60 were annotated in the metabolic pathways, biosynthesis of secondary metabolites, phenylpropanoid biosynthesis, and glycerolipid metabolism; that the highest proportion of differentially expressed metabolites in group P48_VS_S48 were annotated in the metabolic pathways and biosynthesis of secondary metabolites; that the highest proportion of differentially expressed metabolites in group P60_VS_S60 were annotated in the metabolic pathways and biosynthesis of secondary metabolites; and that the highest proportion of differentially expressed metabolites in group S48_VS_S60 were annotated in the biosynthesis of secondary metabolites, flavonoid biosynthesis, and phenylpropanoid biosynthesis.

#### Results of combined differential proteomics and comparative metabolomics

To further determine the effects of different drought stress treatments on the metabolites in the germination stage of castor bean plants and the metabolic pathways in response to drought stress treatments, the differential proteomic analysis of groups P48_VS_P60, P48_VS_S48, P60_VS_S60, and S48_VS_S60 were combined with the results of comparative metabolomic analysis. We used the KEGG metabolic pathways to map the common metabolic pathways of the differentially expressed proteins and differentially expressed metabolites.

##### Results of combined proteomic and metabolomic analysis of group P48_VS_P60

**Fig. 20:**
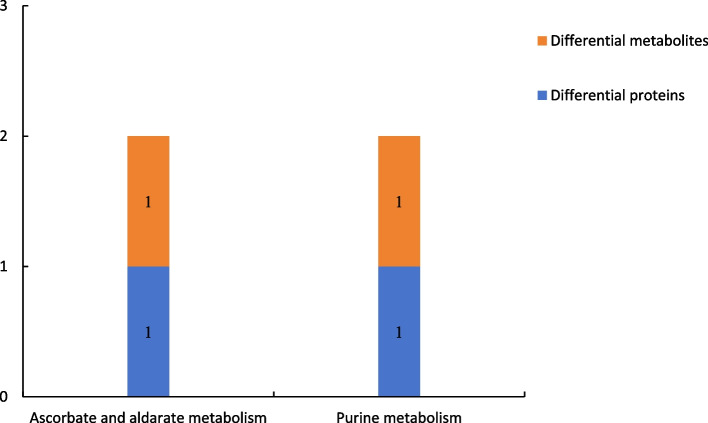
Common metabolic pathways of the differentially expressed proteins and differentially expressed metabolites in group P48_VS_P60. Note: The abscissa represents the metabolic pathways, and the ordinate represents the number of differentially expressed proteins and differentially expressed metabolites simultaneously involved in each metabolic pathway

Figure 20 shows the results of combined analysis of the proteomic and metabolomic data of group P48_VS_P60. The ascorbate and aldarate metabolism and purine metabolism were the common metabolic pathways of the differentially expressed proteins and differentially expressed metabolites of group P48_VS_P60.

##### Results of combined proteomic and metabolomic analysis of group P48_VS_S48

**Fig. 21:**
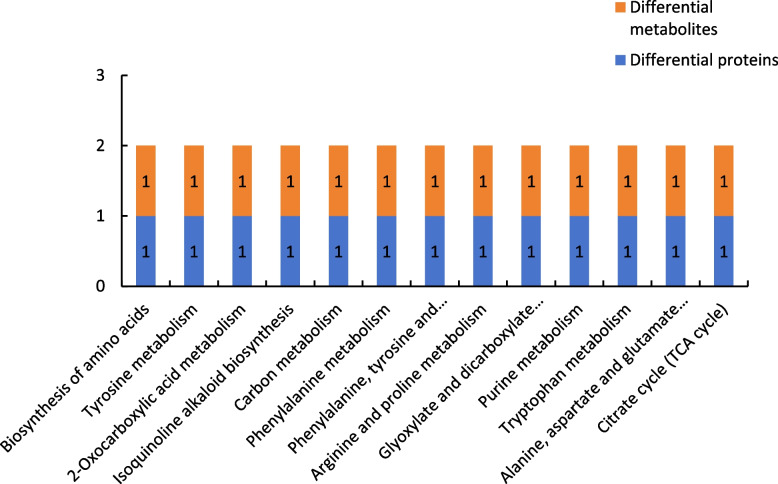
Common metabolic pathways for the differentially expressed proteins and differentially expressed metabolites of group P48_VS_S48. Note: The abscissa represents the metabolic pathways, and the ordinate represents the number of differentially expressed proteins and differentially expressed metabolites simultaneously involved in each metabolic pathway

Figure 21 shows the results of combined analysis of the proteomic and metabolomic data of group P48_VS_S48. The common metabolic pathways of the differentially expressed proteins and differentially expressed metabolites of group P48_VS_S48 included biosynthesis of amino acids, tyrosine metabolism, and 2-oxycarboxylic acid metabolism.

##### Results of combined proteomic and metabolomic analysis of group P60_VS_S60

**Fig. 22:**
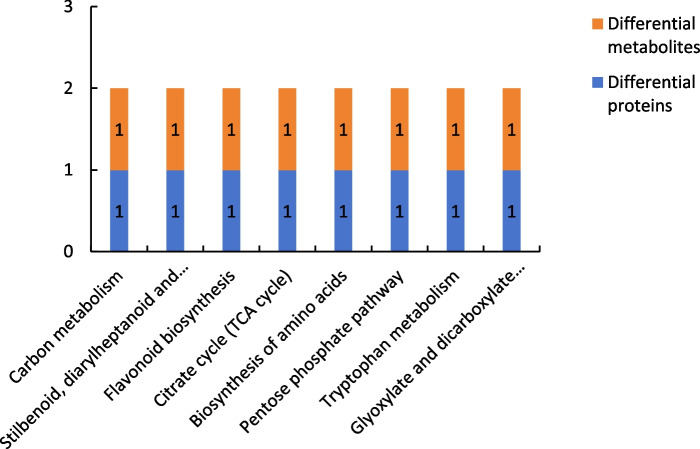
Common metabolic pathways for the differentially expressed proteins and differentially expressed metabolites of group P60_VS_S60. Note: The abscissa represents the metabolic pathways, and the ordinate represents the number of differentially expressed proteins and differentially expressed metabolites simultaneously involved in each metabolic pathway

Figure 22 shows the results of combined analysis of the proteomic and metabolomic data of group P60_VS_S60. The common metabolic pathways of the differentially expressed proteins and differentially expressed metabolites of group P60_VS_S60 included carbon metabolism and stilbenoid, diarylheptanoid, and gingerol biosynthesis.

##### Results of combined proteomic and metabolomic analysis of group S48_VS_S60

The results of combined analysis of the proteomic and metabolomic data of group S48_VS_S60 showed that there were three pathways for differentially expressed proteins in water-treated plants at different times, namely, protein processing in the endoplasmic reticulum, protein export, and ubiquitin-mediated proteolysis. The differentially expressed metabolites of group S48_VS_S60 were mainly involved in the biosynthesis and metabolism of amino acids. The proteomic and metabolomic data did not share any common metabolic pathways.

#### RT-qPCR analysis of drought resistance-related genes

**Fig. 23:**
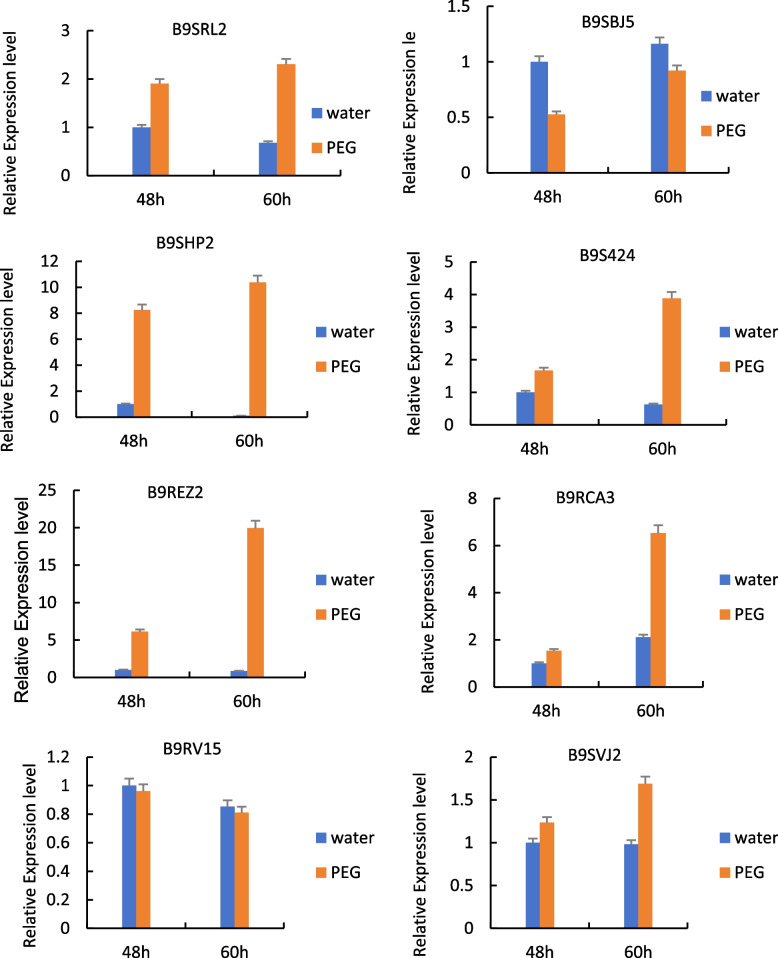
RT-qPCR detection results of the genes encoding some differentially expressed proteins in castor plants in response to drought stress. Note: Different lowercase letters represent significant differences (*P*<0.05)

RT-qPCR analysis was performed on the genes (*RcECP63*, *RcDDX31*, *RcA/HD1*, *RcRANGAP1*, *RcITP*, *RcGST1*, *RcLEAD7*, *RcVTVS13*) encoding eight differentially expressed proteins (B9SRL2, B9SBJ5, B9SHP2, B9S424, B9REZ2, B9RCA3, B9RV15, B9RCA3 B9SVJ2) that might be involved in the drought stress response to study their expression patterns at the transcriptional level. The results are shown in Fig. 23.

### Expression analysis of transient silencing of differential genes in castor bean (*Ricinus communis* L.)

#### Results of phenotyping of leaves

**Fig. 24:**
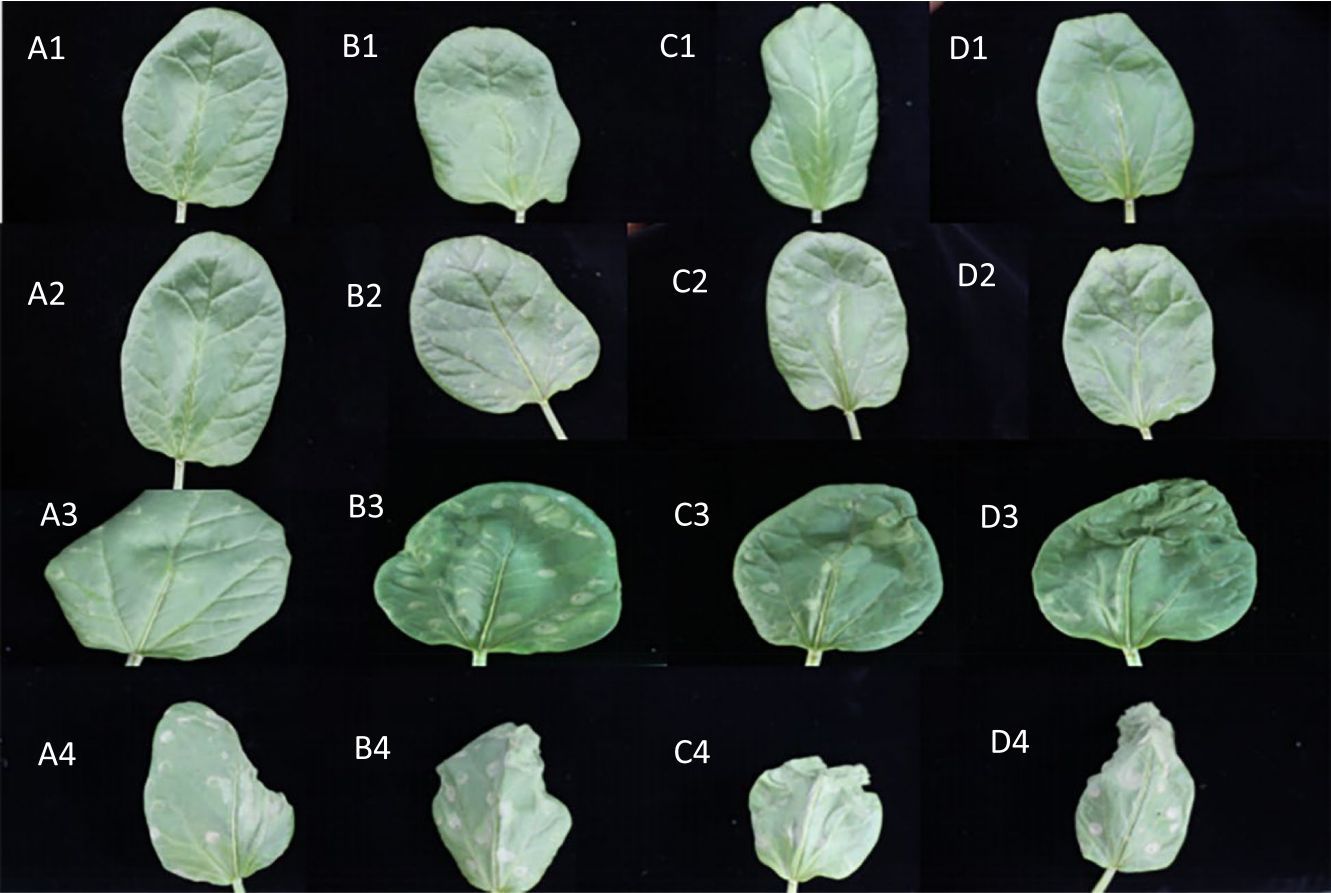
Phenotypic changes of castor bean leaves after transient infection. Note: A1-A4 are the leaf states of wild-type castor leaves treated with 15% PEG 6000 for 0 h, 0 h, 24 h and 48 h respectively; B1-B4 are the leaf states treated with 15% PEG 6000 for 0 h, 24 h and 48 h before and after PTRV-RcECP 63 bacterial solution; C1-C4 are the leaf states treated with 15% PEG 6000 for 0 h, 24 h and 48 h before impregnation with PTRV-RcDDX 31 and after injection of PTRV-RcDDX 31 bacterial solution; D1-D4 are the leaf states treated with 15% PEG 6000 for 0 h, 24 h and 48 h before impregnation with PTRV-RcA/HD1 and after injection of PTRV-RcA/HD1 bacterial solution

Before the stress treatment, castor leaves had good growth and were in a more consistent state; when 15% PEG was treated for 24 h, it could be seen that there was obvious wilting of the leaves and obvious curling occurred at the edges of the leaves; when 15% PEG was treated for 48 h, the loss of water in the leaves was more serious and the curling phenomenon was more obvious, which indicated that at this point in time, after the silencing of the studied genes, *RcECP 63*, *RcDDX 31*, and *RcA/HD1*, the leaves were more intolerant of the drought stress.The result is shown in the Fig. 24.

#### Results of RT-qPCR analysis of differentially expressed genes in leaves

**Fig. 25:**
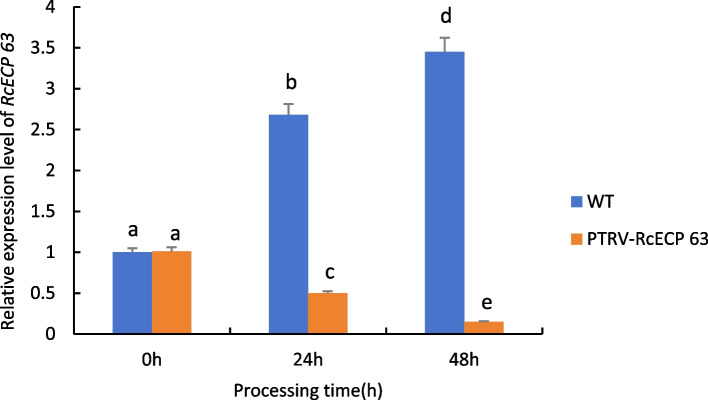
Expression analysis of *RcECP 63* gene transiently infected castor leaves under drought stress. Note: Different lowercase letters represent significant differences(*P* < 0.05)

As can be seen from Fig. 25, when castor plants were subjected to drought stress, the expression of the *RcECP 63* gene in wild-type castor leaves was significantly up-regulated with the increase of drought stress time; whereas the expression of the gene in castor leaves injected with recombinant PTRV-*RcECP 63* bacterial broth under the same treatment conditions all showed a down-regulation trend, which was even more pronounced with the prolongation of time, indicating that the gene was silenced.

**Fig. 26:**
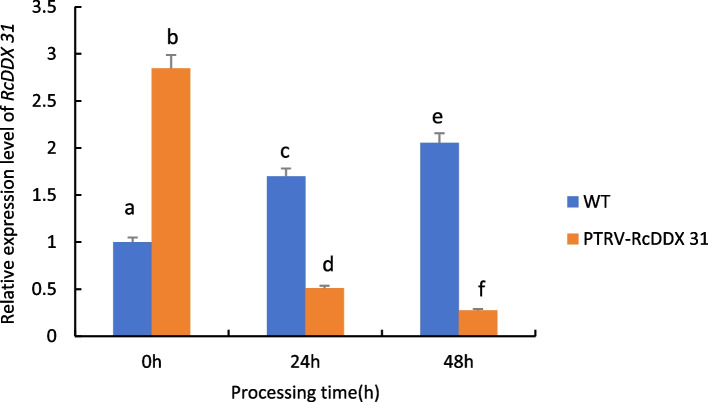
Expression analysis of *RcDDX 31* gene transiently infected castor leaves under drought stress. Note: Different lowercase letters represent significant differences (*P* < 0.05)

As can be seen from Fig. 26, when castor plants were subjected to drought stress, the expression of the *RcDDX 31* gene in wild-type castor leaves showed a significant up-regulation trend with the increase of drought stress time; whereas the expression of the gene in castor leaves injected with recombinant PTRV-*RcDDX 31* bacterial broth under the same treatment conditions showed a significant down-regulation trend, which was even more pronounced with the increase of time, indicating that the gene was silenced.

**Fig. 27:**
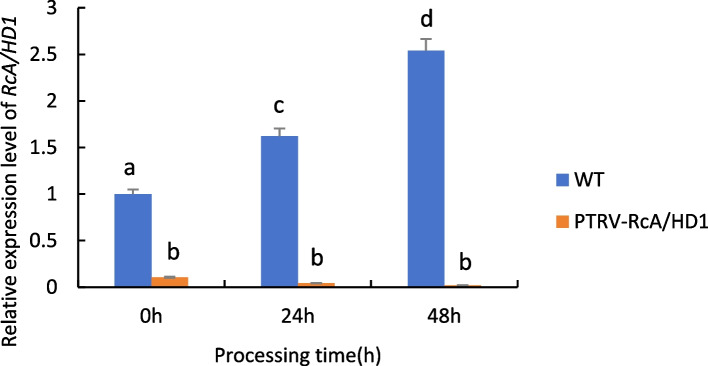
Expression analysis of *RcA/HD1* gene transiently infected castor leaves under drought stress. Note: Different lowercase letters represent significant differences (*P* < 0.05)

As can be seen from Fig. 27, when castor plants were subjected to drought stress, the expression of the *RcA/HD1* gene in wild-type castor leaves was significantly up-regulated with the increase of drought stress time; whereas the expression of the gene in castor leaves injected with recombinant PTRV-*RcA/HD1* bacterial fluids under the same treatment conditions all showed a down-regulation trend, which was even more pronounced with the prolongation of time, indicating that the gene was silenced.

Combining the experimental results of the three sets of data, it can be seen that when the three selected genes were transiently silenced, the expression of the genes showed a significant down-regulation compared to the wild-type control, in which the expression of the *RcA/HD1* gene was less and the decline was more pronounced, which further suggests that the three selected genes are associated with drought resistance in castor bean.

### Functional analysis of differentially expressed genes in *A. thaliana*

#### Vector construction and identification of expression plants

The specific primers were used to amplify the target genes *RcECP 63*, *RcDDX 31* and *RcA/HD1*, respectively, and *Nco*I single enzyme digestion was performed on the pairs of heterologous expression vector pCAMBIA1305.2, and the results were all available for the next step of the experiment. The constructed vector plasmid was transformed into Agrobacterium *GV3101* strain. The recombinant Agrobacterium sap was used to dip Arabidopsis thaliana (wild type and its mutants Col-0, *atecp63*, *atddx31-1*, *atddx31-2*, *ata/hd1*) using the flower dipping method, and overexpression-resistant plants *ECP 63-OE*, *DDX 31-OE*, *A/HD1-OE* and back-expression-resistant plants *ECP 63* -The transgenic plants were analysed at the molecular and biological levels to determine the gene functions.

**Fig. 28:**
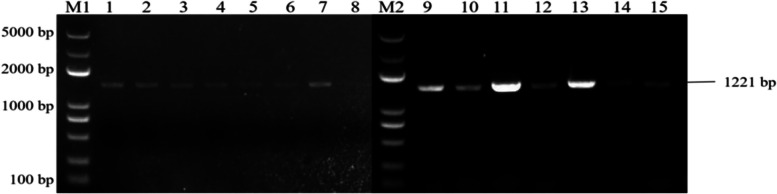
Results of PCR identification of the resistant *Arabidopsis thaliana* plants with overexpression and complementary expression of *RcECP63*. Note: M1 and M2: DL 5000 Marker; 1-8: PCR identification results of the resistant complementary-expression *A. thaliana* plant *ECP63-GR*. 9-15: PCR identification results of the resistant overexpression *A. thaliana* plant* ECP63-OE*

**Fig. 29:**
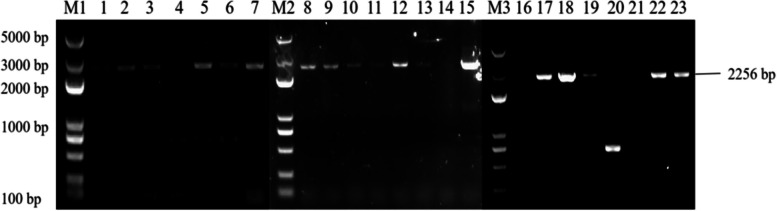
Results of PCR identification of the resistant *Arabidopsis thaliana* plants with overexpression and complementary expression of *RcDDX31*. Note: M1, M2, and M3: DL 5000 Marker; 1-7: PCR identification results of the resistant complementary-expression *A. thaliana* plant *DDX31-GR1*; 8-15: PCR identification results of the resistant complementary-expression *A. thaliana* plant *DDX31-GR2*; 16-23: PCR identification results of the resistant overexpression *A. thaliana* plant *DDX31-OE*

**Fig. 30:**
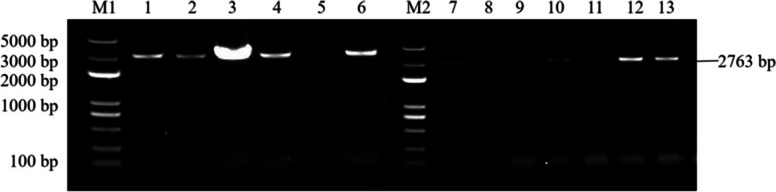
Results of PCR identification of the resistant *Arabidopsis thaliana* plants with overexpression and complementary expression of *RcA/HD1*. Note: M1 and M2: DL 5000 Marker; 1-6: PCR identification results of the resistant complementary-expression *A. thaliana* plant *A/HD1-GR*; 7-13: PCR identification of the resistant overexpression *A. thaliana* plant *A/HD1-OE*

PCR identification of overexpressing Arabidopsis plants and back-expressing Arabidopsis plants showed that seven overexpressing Arabidopsis *ECP 63-OE*, five overexpressing Arabidopsis *DDX 31-OE*, and five overexpressing Arabidopsis *A/HD1-OE* plants were obtained, as well as seven back-expressing Arabidopsis *ECP 63-GR*, seven back-expressing Arabidopsis *DDX31-GR1*, and four back-expressing Arabidopsis *A/HD1-GR*. The results are shown in Figs. 28, 29 and 30.

#### RT-qPCR identification results of overexpression and complementary- expression transgenic *A. thaliana* plants

RNA was extracted from overexpressing and back-expressing Arabidopsis transgenic plants, and the samples were analysed by RT-qPCR using RNA reverse-transcribed cDNA as a template.

**Fig. 31:**
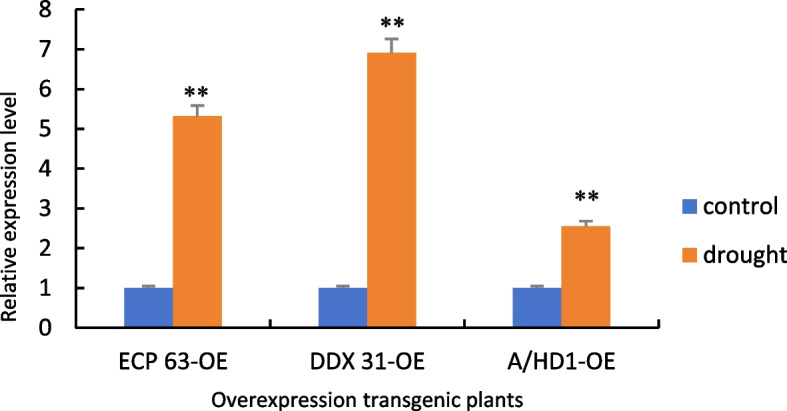
RT-qPCR results of positive overexpression *Arabidopsis thaliana* plants. Note: *: 0.01 <*P*≤0.05, **: 0<*P*≤0.01

Figure 31 shows the RT-qPCR analysis of the samples with the reverse-transcribed cDNA of the overexpression plants as the template. Compared with the controls, the gene expression levels of the overexpression plants were significantly increased under drought stress. This indicates that due to changes in the environment, the transcripts of genes are significantly increased during the transcription process, which allows the plants to respond to drought stress.

**Fig. 32:**
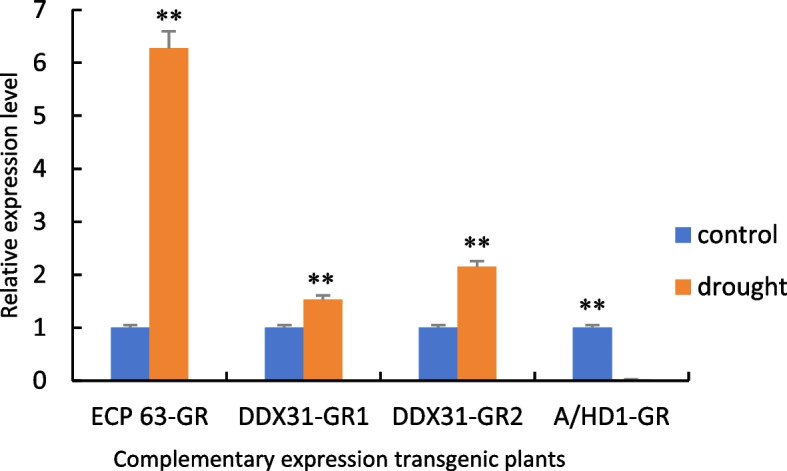
RT-qPCR results of positive complementary-expression *Arabidopsis thaliana* plants. Note: *: 0.01 <*P*≤0.05, **: 0<*P*≤0.01

Figure 32 shows the RT-qPCR analysis of the samples with the cDNA obtained from the reverse transcription of the complementary-expression plants as the template. Compared with the controls, the gene expression levels of the four types of plants with complementary expression of *RcECP63* and *RcDDX31* were significantly increased under drought stress, while the gene expression levels of the plants with complementary expression of RcA/HD1 were significantly reduced under drought stress. These results indicate that the plants with the complemented deletion mutant of RcA/HD1 had an insignificant response to drought stress.

#### Analysis of the tolerance of overexpression and complementary-expression *A. thaliana* plants to drought stress

##### Analysis of the response of the *RcECP63* gene to drought stress

**Fig. 33:**
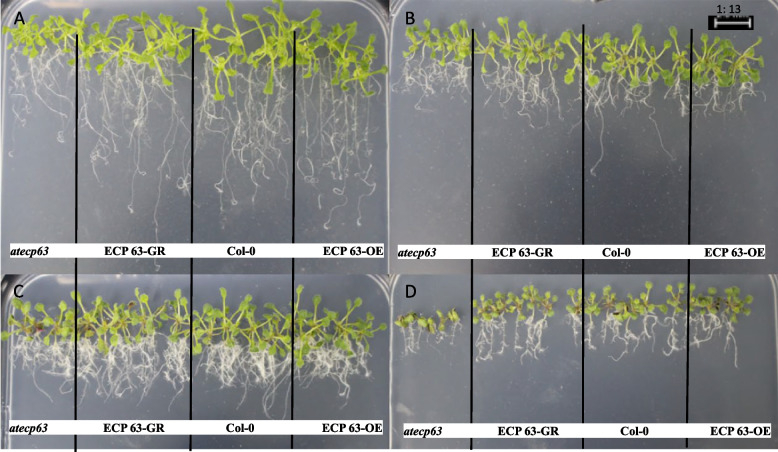
Effect of different concentrations of mannitol on *atecp63*, *ECP 63-GR*, Col-0, and *ECP 63-OE*. Note: A-D represent the mannitol medium containing 0 mmol/L, 100 mmol/L, 200 mmol/L, and 300 mmol/L mannitol, respectively

**Fig. 34:**
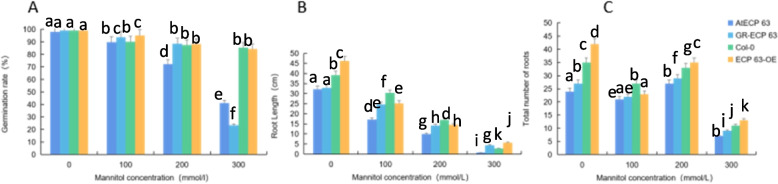
Germination rate, root length, and number of lateral roots of the four types of *Arabidopsis thaliana* plants by *RcECP63* genotype. Note: Figure A shows the germination rate of *A. thaliana* under drought stress treatments with different mannitol concentrations; Figure B shows the root lengths of *A. thaliana* plants treated with different mannitol concentrations; Figure C shows the numbers of lateral roots of *A. thaliana* plants treated with different mannitol concentrations. Different lowercase letters represent significant differences (*P*<0.05)

We analyzed the effects of drought stress treatments with different mannitol concentrations on the growth and development of mutant, complementary-expression, wild-type, and overexpression *A. thaliana (atecp63*, *ECP63-GR*, Col-0, and *ECP63-OE*) and analyzed the germination rates, root lengths, and numbers of lateral roots of *A. thaliana* plants under drought stress treatments with different mannitol concentrations. The results are shown in Figs 33 and 34. The plants without mannitol treatment grew normally. The drought stress significantly changed the morphology of *A. thaliana*. When *A. thaliana* plants were treated with 100 mmol/L mannitol, the overexpression *A. thaliana* plants showed significantly shorter root lengths and significantly more lateral roots than the controls. When *A. thaliana* plants were treated with 200 mmol/L mannitol, the number of lateral roots was still significantly increased. When *A. thaliana* plants were treated with 300 mmol/L mannitol, the number of lateral roots and the length of lateral roots were significantly downregulated, and the abaxial epidermis of the leaves of all four types of *A. thaliana* plants turned reddish-brown, especially in the mutant *A. thaliana* plants, and the *A. thaliana* plants suffered severe inhibition of their growth and development and severe damage.

##### Analysis of the response of the *RcDDX31* gene to drought stress

**Fig. 35:**
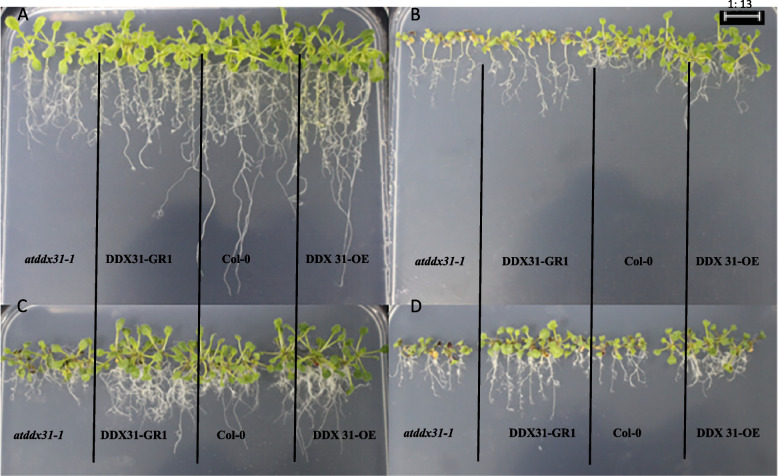
Effect of different mannitol concentration on *atddx31-1*, *DDX31-GR1*, Col-0, and *DDX31-OE*. Note: A-D represents the mannitol medium containing 0 mmol/L, 100 mmol/L, 200 mmol/L, and 300 mmol/L mannitol, respectively

**Fig. 36:**
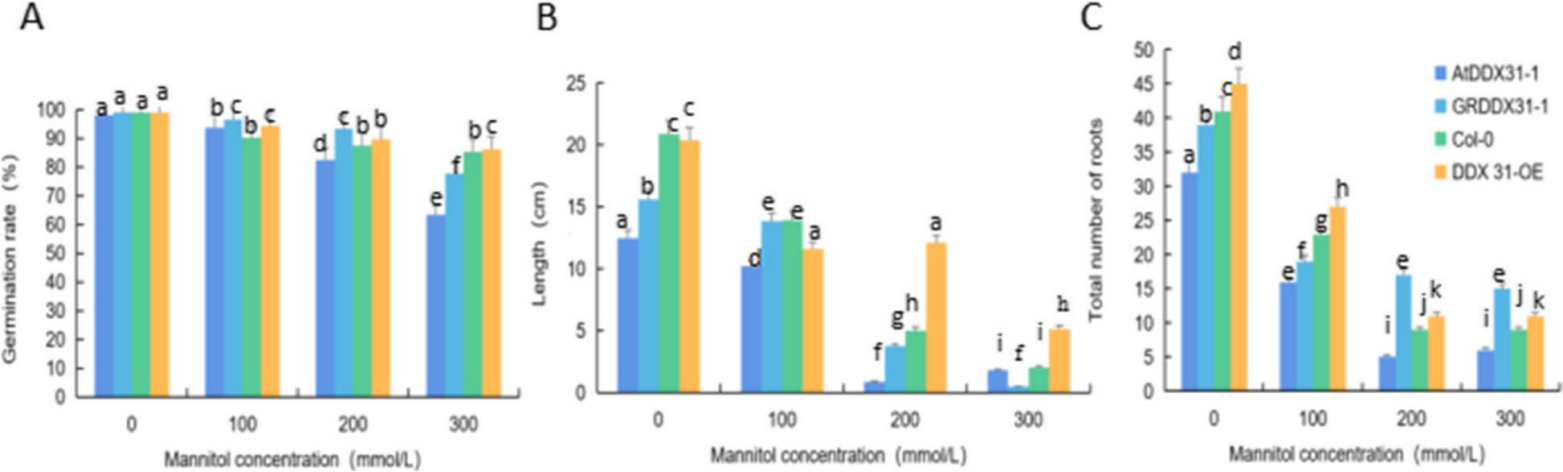
Analysis results of the germination rates, root lengths, and numbers of lateral roots of the four types of *Arabidopsis thaliana* corresponding to the *RcDDX31* mutant *atddx31-1*. Note: Figure A shows the germination rates of *A. thaliana* drought stress treatments with different mannitol concentrations. Figure B shows the root lengths of *A. thaliana* plants under drought stress treatments with different mannitol concentrations. Figure C shows the numbers of lateral roots of *A. thaliana* plants under drought stress treatments with different mannitol concentrations. Different lowercase letters represent significant differences (*P*<0.05)

We analyzed the effects of drought stress treatments with different mannitol concentrations on the growth and development of mutant, complementary-expression, wild-type, and overexpression A. thaliana (*atddx31-1*, *DDX31-GR1*, Col-0, and *DDX31-OE*) and analyzed the germination rates, root lengths, and numbers of lateral roots of *A. thaliana* plants under drought stress treatments with different mannitol concentrations. The results are shown in Figs. 35 and 36. *A. thaliana* grew normally without stress treatment, and the complementary-expression plant *DDX31-GR1* had the longest root length. Drought stress changed the morphology of *A. thaliana* significantly. When *A. thaliana* plants were treated with 100 mmol/L mannitol, the root length was significantly shorter, and the number of lateral roots significantly increased compared with that of the controls. When *A. thaliana* plants were treated with 200 mmol/L mannitol, the number of lateral roots was significantly increased. When *A. thaliana* plants were treated with 300 mmol/L mannitol, the number of lateral roots was even less, and the length of lateral roots were even shorter, and the abaxial epidermis of the leaves of all four types of *A. thaliana* plants turned reddish-brown, which indicates that the tolerance of *A. thaliana* to mannitol was reduced at this concentration.

**Fig. 37:**
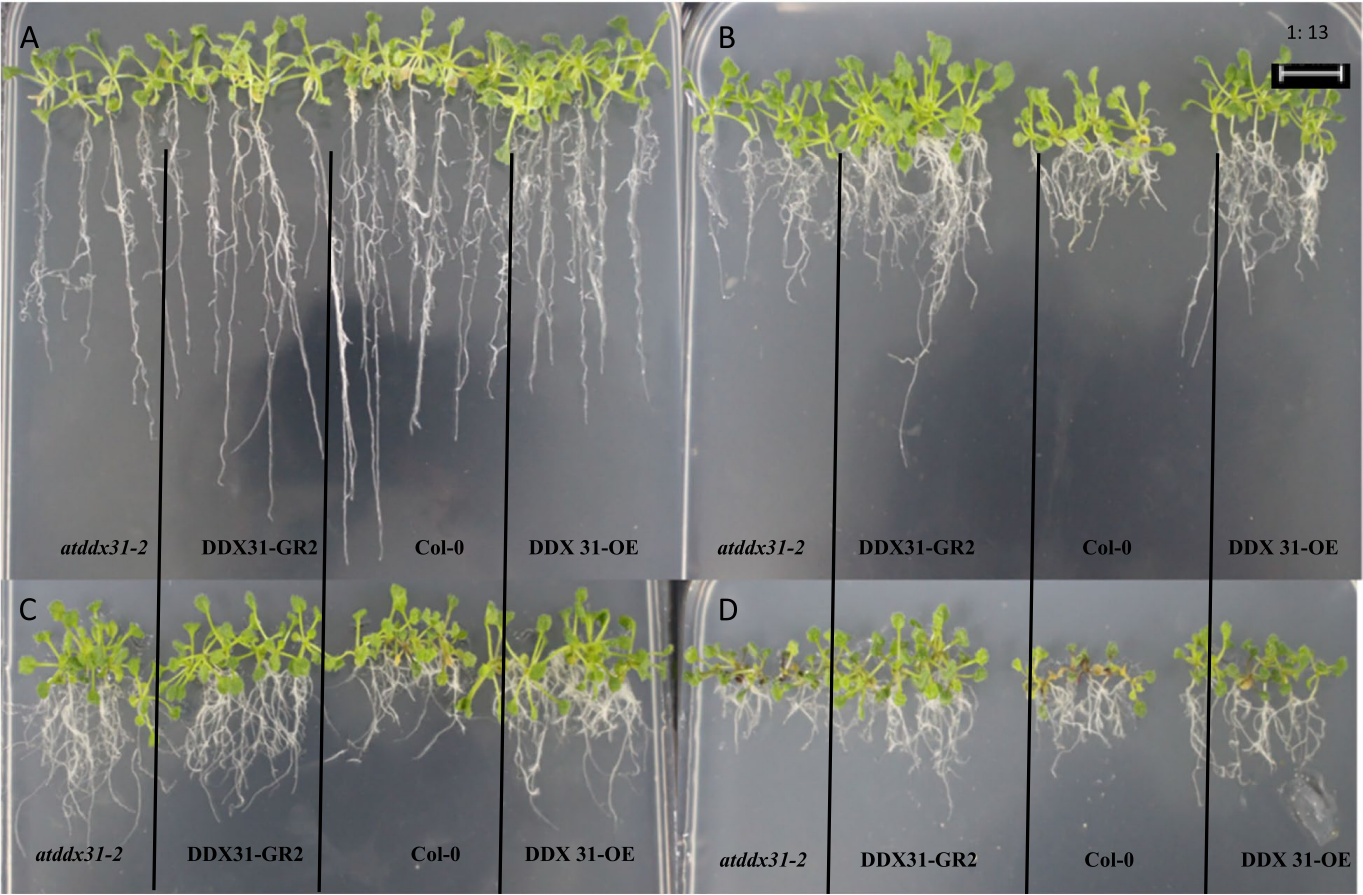
Effects of different mannitol concentrations on *atddx31-2*, *DDX31-GR2*, Col-0, and *DDX31-OE*. Note: A-D represent the mannitol medium containing 0 mmol/L, 100 mmol/L, 200 mmol/L, and 300 mmol/L mannitol, respectively

**Fig. 38:**
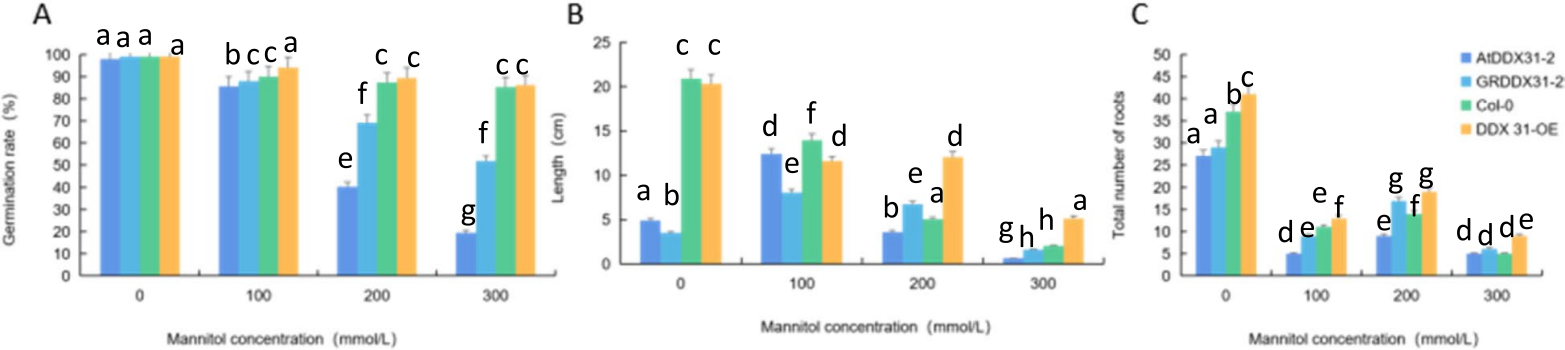
Analysis results of germination rates, root lengths, and numbers of lateral roots of the four types of *Arabidopsis thaliana* plants corresponding to the *RcDDX31* mutant *atddx31-2*. Note: Figure A shows the germination rates of *A. thaliana* plants under drought stress treatments with different mannitol concentrations. Figure B shows the root lengths of *A. thaliana* plants under drought stress treatments with different mannitol concentrations. Figure C shows the number of lateral roots of *A. thaliana* plants under drought stress treatments with different mannitol concentrations. Different lowercase letters represent significant differences (*P*<0.05)

We analyzed the effects of drought stress treatments with different mannitol concentrations on the growth and development of mutant, complementary-expression, wild-type, and overexpression *A. thaliana* plants (*atddx31-2*, *DDX31-GR2*, Col-0, and *DDX31-OE*) and analyzed the germination rates, root lengths, and numbers of lateral roots of *A. thaliana* plants under drought stress treatments with different mannitol concentrations. The results are shown in Figs. 37 and 38. The plants grew normally without stress treatment. Drought stress changed the morphology of *A. thaliana* significantly. When *A. thaliana* plants were treated with 100 mmol/L mannitol, the numbers of lateral roots in the mutant and complementary-expression plants were less increased, and a small number of leaves were chlorotic. When *A. thaliana* plants were treated with 200 mmol/L mannitol, the overall growth was better, and the number and length of lateral roots further increased, and the leaves of mutant and complementary-expression plants turned chlorotic or reddish-brown. When *A. thaliana* plants were treated with 300 mmol/L mannitol, the number and length of lateral roots were less than when they were treated with 100 mmol/L and 200 mmol/L mannitol, and all leaves turned reddish-brown, but the overexpression and complementary-expression plants had more and longer lateral roots than the wild-type and mutant plants, which indicates that the response of this gene to drought stress was mainly manifested in the number and length of lateral roots.

##### Analysis of the response of the *RcA/HD1* gene to drought stress

**Fig. 39:**
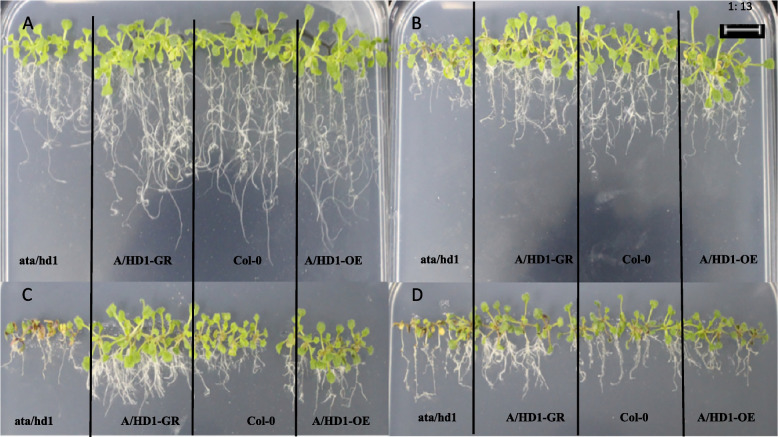
Effect of different mannitol concentrations on ata/hd1, *A/HD1-GR*, Col-0, and *A/HD1-OE*. Note: A-D represents the mannitol medium containing 0 mmol/L, 100 mmol/L, 200 mmol/L, and 300 mmol/L, respectively

**Fig. 40:**
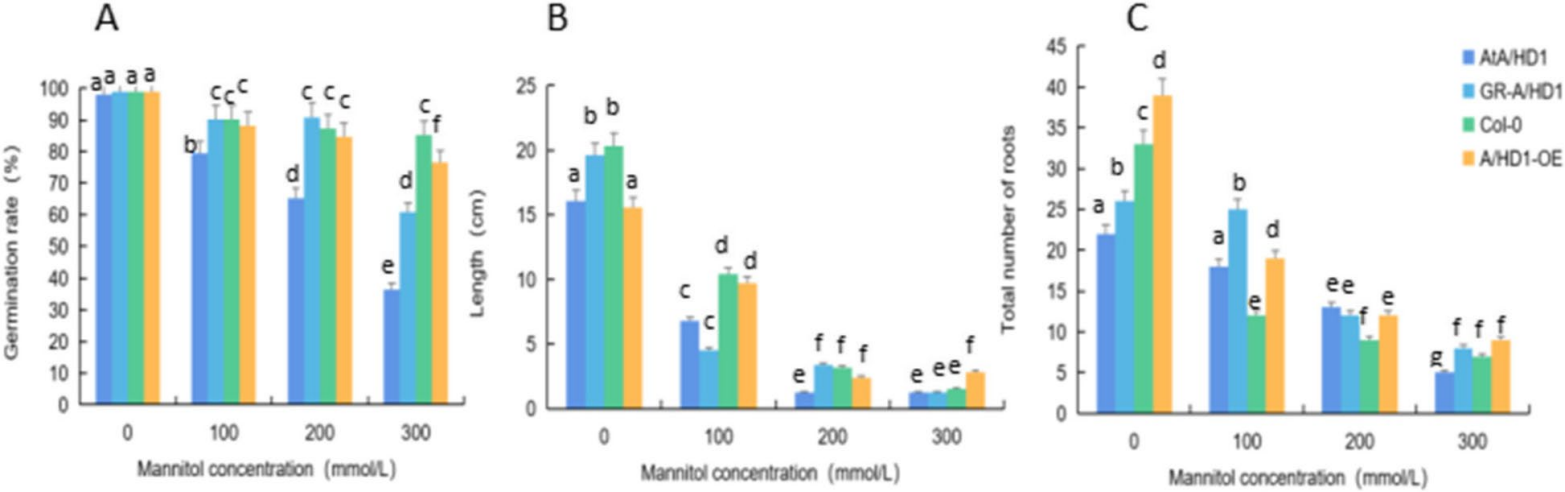
Analysis results of the germination rates, root lengths, and number of lateral roots of the four types of *Arabidopsis thaliana* corresponding to *RcA/HD1*. Note: Figure A shows the germination rates of *A. thaliana* plants under drought stress treatments with different mannitol concentrations. Figure B shows the root lengths of *A. thaliana* plants under drought stress treatments with different mannitol concentrations. Figure C shows the numbers of lateral roots of *A. thaliana* plants under drought stress treatments with different mannitol concentrations. Different lowercase letters represent significant differences (*P*<0.05)

To study the response mechanism of the *RcA/HD1* gene to drought stress in *A. thaliana* plants, we phenotypically identified *ata/d1*, *A/HD1-GR*, Col-0, and *A/HD1-OE* and analyzed the germination rates, root lengths, and numbers of lateral roots under drought stress treatments with different mannitol concentrations. The results are shown in Figs. 39 and 40. *A. thaliana* plants grew normally without stress treatment. When *A. thaliana* plants were treated with 100 mmol/L mannitol, the four types of plants had short root lengths and a limited increase in root number. When *A. thaliana* plants were treated with 200 mmol/L mannitol, they had shorter root lengths but more lateral roots and chlorotic or reddish-brown leaf abaxial epidermis, and the overexpression and complementary-expression plants had more later roots and longer root lengths. When *A. thaliana* plants were treated with 300 mmol/L mannitol, they showed weaker overall growth, shorter lateral roots, and reddish-brown leaf abaxial epidermis, but the overexpression and complementary-expression plants had more lateral roots. These findings indicated that the response of this gene to drought stress was mainly manifested in the number of roots and the degree of root elongation.

##### Relevant physiological indicators of *A. thaliana* under drought stress

**Fig. 41:**
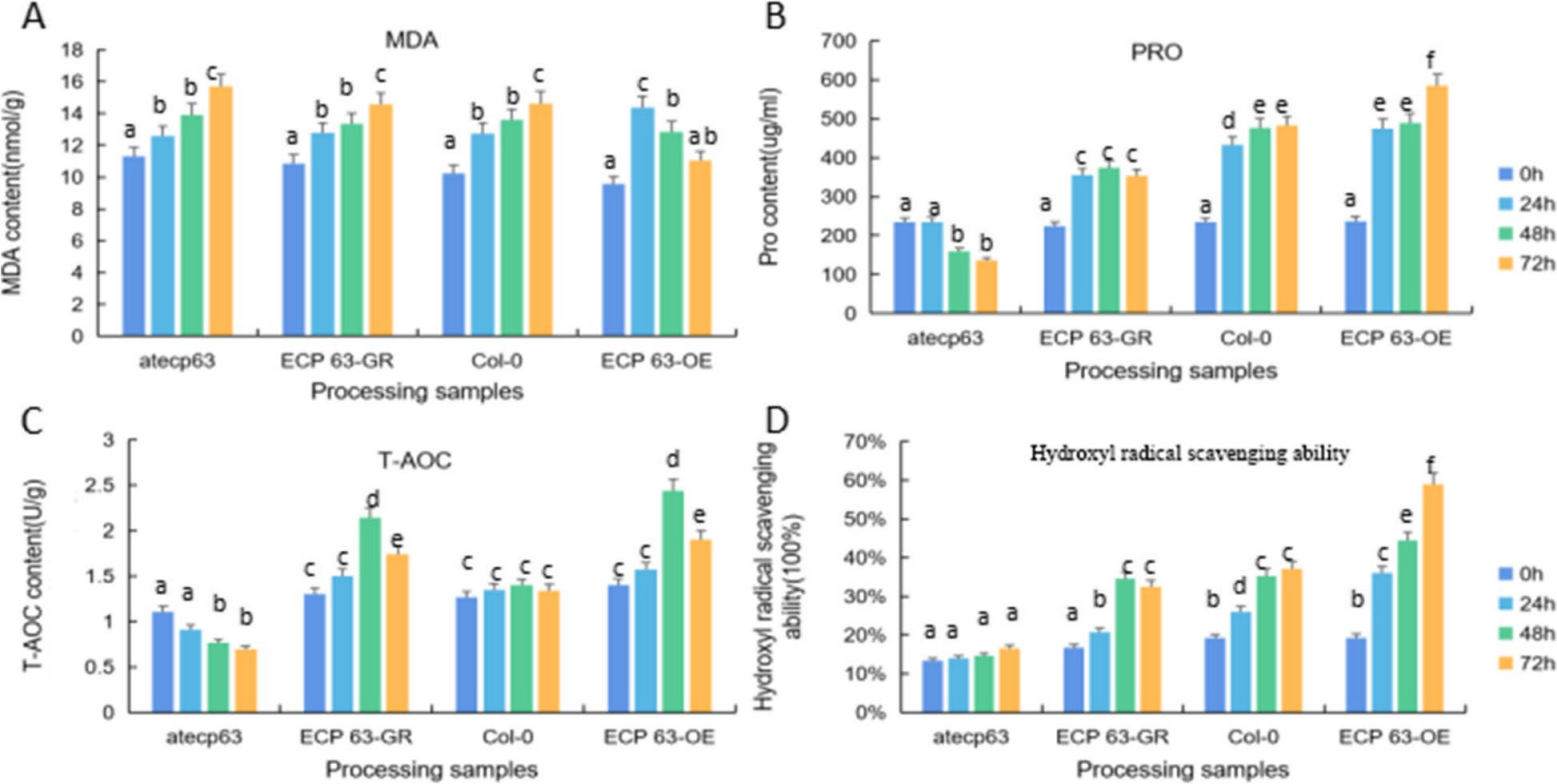
Physiological indicators of four types of *Arabidopsis thaliana* plants with different *RcECP63* genotypes under drought stress treatment with PEG 6000. Note: A: MDA measurement results of *atecp63*, *ECP63-GR*, Col-0, and *ECP63-OE* at 0 h, 24 h, and 48 h. B: PRO measurement results of *atecp63*, *ECP63-GR*, Col-0, and *ECP63-OE* at 0 h, 24 h, and 48 h. C: T-AOC measurement results of *atecp63*, *ECP63-GR*, Col-0, and *ECP63-OE* at 0 h, 24 h, and 48 h. D: Measurement results of hydroxyl radical scavenging ability of *atecp63*, *ECP63-GR*, Col-0, and *ECP63-OE* at 0 h, 24 h, and 48 h. different lowercase letters indicate significant differences (*P*<0.05)

**Fig. 42:**
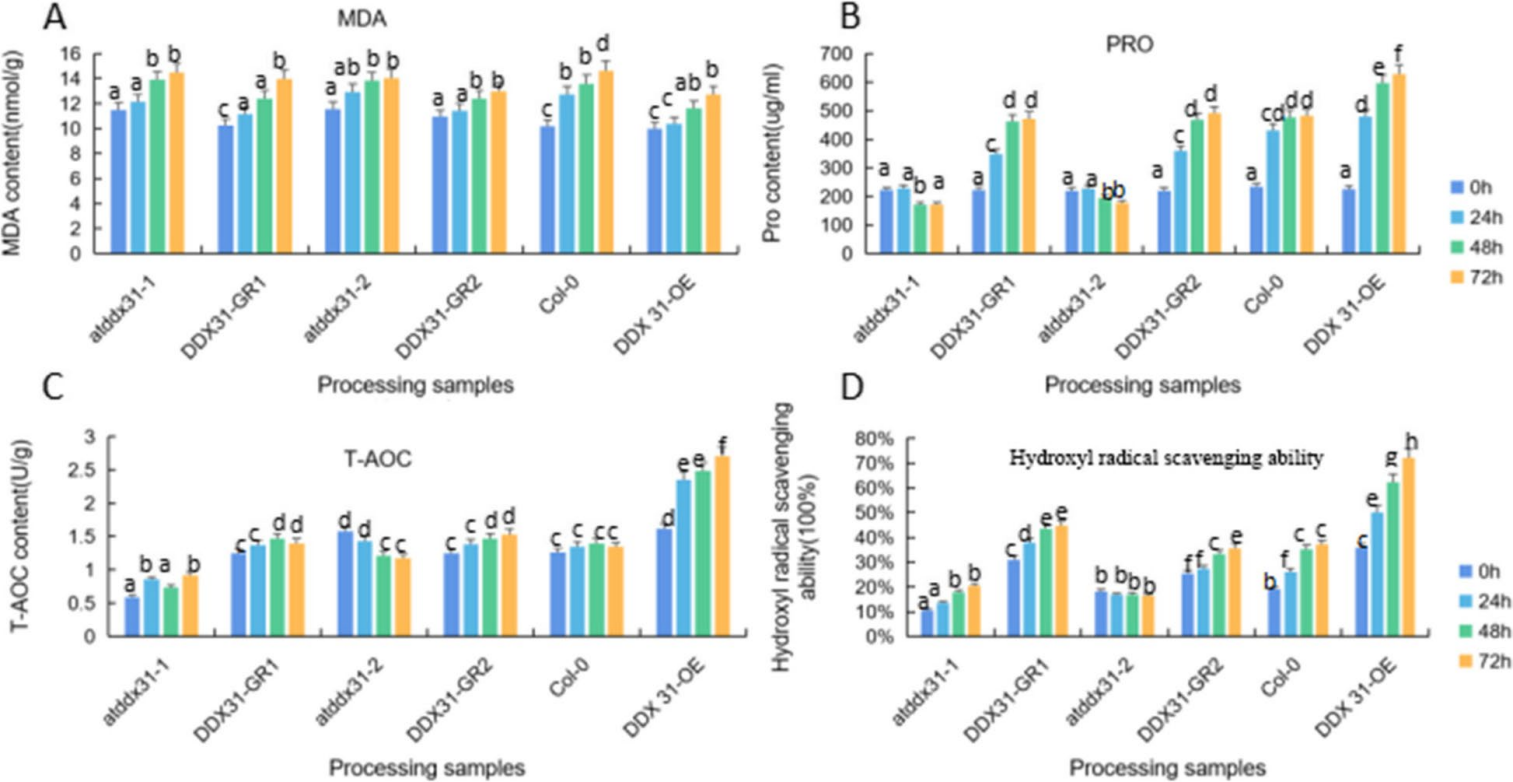
Physiological indicators of four types of Arabidopsis thaliana plants corresponding to the *RcDDX31* gene under PEG 6000 stress. Note: A: MDA concentrations of *atddx31-1*, *DDX31-GR1*, *atddx31-2*, *DDX31-GR2*, Col-0, and *DDX31-OE* at 0 h, 24 h, and 48 h. B: Proline concentrations of *atddx31-1 DDX31-GR1*, *atddx31-2*, *DDX31-GR2*, Col-0, and *DDX31-OE* at 0 h, 24 h, and 48 h. C: T-AOC of *atddx31-1*, *DDX31-GR1*, *atddx31-2*, *DDX31-GR2*, Col-0, and *DDX31-OE* at 0 h, 24 h, and 48 h. D: Hydroxyl radical scavenging ability of *atddx31-1*, *DDX31-GR1*, *atddx31-2*, *DDX31-GR2*, Col-0, and *DDX31-OE* at 0 h, 24 h, and 48 h. Lowercase letters represent significant differences (*P*<0.05)

**Fig. 43:**
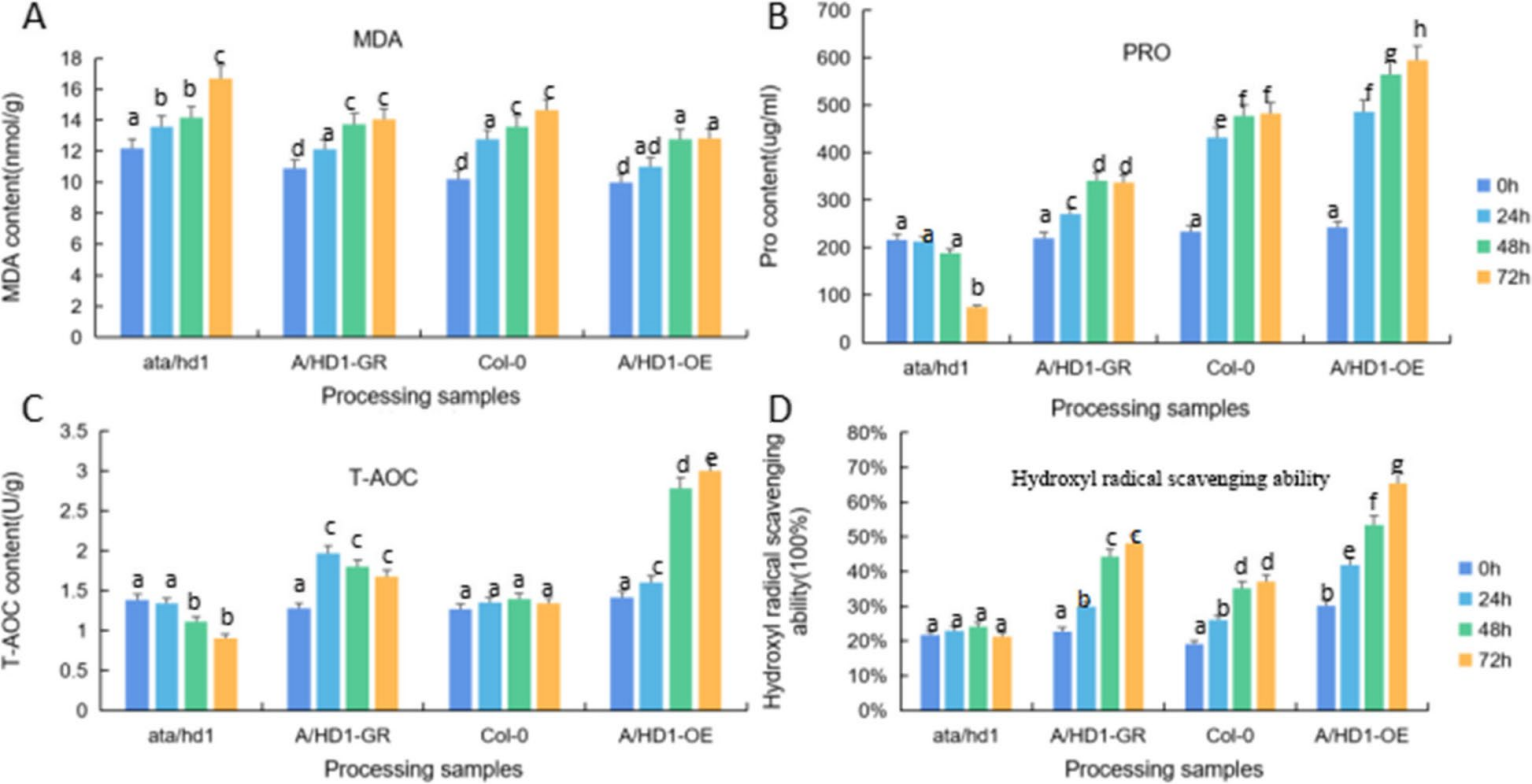
Physiological indicators of four types of *Arabidopsis thaliana* according to the *RcA/HD1* gene under PEG 6000 stress. Note: A: MDA concentration of *ata/d1, A/HD1-GR*, Col-0, and *A/HD1-OE* at 0 h, 24 h, and 48 h. B: Proline concentration of *ata/d1, A/HD1-GR*, Col-0, and *A/HD1-OE* at 0 h, 24 h, 48 h. C: T-AOC of *ata/d1, A/HD1-GR*, Col-0, and A/HD1-OE at 0 h, 24 h, and 48 h. D: Hydroxyl radical-scavenging ability of *ata/d1, A/HD1-GR*, Col-0, and *A/HD1-OE* at 0 h, 24 h, and 48 h. different lowercase letters represent significant differences (*P*<0.05)

We measured the physiological indicators (MDA, proline, T-AOC, and hydroxyl radical scavenging ability) of the *A. thaliana* plants treated with 10% PEG 6000 (drought stress) for 0 h, 24 h, 48 h, and 72 h, with water-treated *A. thaliana* plants as controls. The measurement results are shown in Figs. 41, 42 and 43.

Figure 41 shows the measurement results of MDA content, proline content, T-AOC content, and hydroxyl radical scavenging ability of *A. thaliana* plants with different *RcECP63* genotypes at different treatment durations. The results showed that as the stress duration increased, the proline content, hydroxyl radical scavenging ability, and T-AOC content of *ECP63-GR*, Col-0, and *ECP63-OE* all significantly increased, while the proline and T-AOC contents of the deletion mutant significantly increased. The MDA and T-AOC contents in the overexpression *A. thaliana* plants first increased and then decreased. We speculate that the response mechanism of the *RcECP63* gene to drought stress is time-dependent and that its activity gradually decreases with increasing treatment duration.

Figure 42 shows the measurement results of MDA content, proline content, T-AOC content, and hydroxyl radical scavenging ability of *A. thaliana* plants of different *RcDDX31* genotypes at different treatment durations. Compared with other types, the overexpression plants had lower MDA contents and the least increase in MDA content. As the stress duration increased, the proline content and hydroxyl radical-scavenging ability increased significantly in the complementary-expression, wild-type, and overexpression plants but remained unchanged or even decreased in the mutant plants, while the T-AOC content showed an overall stable trend in *A. thaliana* plants but a significant increase in the overexpression plants, which was consistent with the MDA measurement results. The results indicate that overexpressing *RcDDX31* enhances the plant drought resistance, which further solidifies the role of *RcDDX31* as a drought resistance gene.

Figure 43 shows the measurement results of MDA content, proline content, T-AOC content, and hydroxyl radical scavenging ability of *A. thaliana* plants of different *RcA/HD1* genotypes at different treatment durations. The results showed that as the drought stress increased, the proline content, T-AOC content, and hydroxyl radical scavenging ability of the complementary-expression, wild-type, and overexpression plants increased overall, and these indicators were significantly upregulated in overexpression plants. As the drought stress increased, the MDA content, which reflects the degree of damage, in the complementary-expression, wild-type, and overexpression plants increased but was lower than that in the overexpression plants, and the increment in MDA content in these three types of plants was less than that in the overexpression plants. These results indicate that the transgenic plants responded to external drought stress to adapt to the unfavorable living environment, revealing that *RcA/HD1* has drought resistance function.

The above results indicate that overexpressing *RcECP63*, *RcDDX31*, and *RcA/HD1* can make plants more tolerant to drought stress, have a protective effect on plants, reduce the degree of damage to plants, and enhance their antioxidant activity.

## Methods

### Physiological studies on the response of castor embryos to drought stress during germination

The data analysis for each of the following physiological index measurements was carried out using the software SPSS version 19.0 for independent samples t-test, and bar charts were plotted according to the analysis results using WPS software and marked with the significance of differences between samples.

#### Measurement of Catalase (CAT) activity

For experimental details see the determination by Yang-Hu Sima [9].

#### Measurement of Superoxide dismutase (SOD) activity

For experimental details see the determination by A García-Triana [10].

#### Measurement of Peroxidase (POD) activity

For experimental details see the determination by Wang L [11].

#### Measurement of Glutathione S-transferase (GST) activity

For experimental details see the determination by Guo N [12].

#### Measurement of Total antioxidant capacity (T-AOC) capacity

For experimental details see the determination by Naheed Z [13].

#### Measurement of Malondialdehyde (MDA) activity

For experimental details see the determination by SE Castrejón [14].

#### Measurement of Hydrogen peroxide (H2O2) activity

For experimental details see the determination by Yang Hu Sima [9].

#### Measurement of Proline (Pro) activity

For experimental details see the determination by Vieira S M [15].

#### Determination of hydroxyl radical scavenging capacity

For experimental details see the determination by Jingang W [16].

### Differential proteomics and widely targeted metabolomic analyses of castor bean embryos at the germination stage

#### Differential proteomics

The experimental samples were sent to company for protein extraction, trypsin digestion, iTRAQ labeling, high-performance liquid chromatography (HPLC) fractionation, and liquid chromatography‒mass spectrometry (LC‒MS) analysis, protein characterization, database search, and functional analysis. Proteins with significant differences in expression were selected, and proteins with significant differences in drought-related changes were selected from the differentially upregulated proteins.

#### Widely targeted metabolomics

The experimental samples were sent to company for sample preparation, screening of differentially expressed metabolites, and data analysis. The enriched metabolic pathways were analyzed to find the differentially expressed metabolites during drought stress. The metabolic pathways responsible for the changes in metabolite levels and their variation patterns were studied.

#### Combined proteomic and metabolomic analysis

The common metabolic pathways of differentially expressed proteins and differentially expressed metabolites in the same treatment group were identified by differential proteomics and widely targeted metabolomic analysis of the Kyoto Encyclopedia of Genes and Genomes (KEGG)-associated metabolic pathways.

#### RT-qPCR validation of drought resistance-related genes

The RNA of castor bean embryos was extracted, measured for its OD_260_/OD_280_ ratio, and then separated by 1% agarose gel electrophoresis. The RNA solution was reverse-transcribed to obtain cDNA. Eight differentially expressed proteins were selected through differential proteomics, and the mRNAs encoding these proteins were submitted to RT-qPCR analysis to study their expression patterns at the transcriptional level. Expression was normalized to castor bean 18S rRNA gene as an internal reference (Supplementary Table 1).

RT-qPCR was performed using the GoTaq® qPCR and RT-qPCR Systems. The qPCR mixes are shown in Supplementary Table 2. Three biological replicates of each experimental material were used. The average C_T_ was taken to calculate the relative expression levels of the genes using the 2^-ΔΔCт^ method (Supplementary Table 2).

After RT-qPCR of genes encoding differentially expressed proteins, all data were analyzed with SPSS version 19.0 software using independent-sample t test.

### Expression analysis of transient silencing of differential genes in castor bean (*Ricinus communis* L.)

The fusion expression vectors PTRV2-*RcECP 63*, PTRV2-*RcDDX 31* and PTRV2- *RcA/HD1* were constructed, and the three target gene fragments were ligated to the PTRV2 vector using the seamless cloning method, respectively, and the constructed silencing vector was transformed into Agrobacterium tumefaciens (OD600=0.6-1.0). The bacterial solution was resuspended and injected into the grate leaves, cultured for 4 d and then treated with 15% PEG 6000 for drought stress, and wild-type castor leaves were used as the control for RNA extraction and RT-qPCR verification.

### Functions of differentially expressed genes in *A. thaliana*

#### Gene cloning and vector linearization

The coding sequences of the genes *RcECP63*, *RcDDX31*, and *RcA/HD1* were ligated into the expression vector pCAMBIA-1305.2. The restriction enzyme sites on the gene and on the expression vector pCAMBIA1305.2 were analyzed, and the restriction endonuclease *Nco*I was finally selected*.* The primers for seamless cloning of the target genes *RcECP63*, *RcDDX31*, and *RcA/HD1* were designed by using the native software CE Design V1.03 (Supplementary Table 3). The three genes were amplified by PCR using Premix PrimeSTAR HS (Supplementary Table 4). The reaction conditions were 94 °C for 5 min; 40 cycles of 94 °C for 45 s, T_m_ for 1 min 30 s to 2 min 30 s, 72 °C for 10 min; and hold at 4 °C. After the reaction, the PCR products were detected using 1% agarose gel electrophoresis.

The vector pCAMBIA1305.2 was cleaved using the restriction endonuclease *Nco*I recognizing a single restriction enzyme site to obtain a linearized vector (Supplementary Table 5). The reaction conditions were 37 °C in a water bath for 3 h. After the reaction, the enzyme digestion products were detected by 1% agarose gel electrophoresis.

#### Ligation of the target genes with the heterologous expression vector

The PCR products and enzyme digestion products of the three target genes that were correctly detected by electrophoresis were recovered for future use. Several target gene fragments and heterologous expression vector fragments were ligated using a seamless cloning kit, and the obtained recombinant vector was used in subsequent experiments (Supplementary Table 6). The reaction condition was 50 °C for 1 h.

#### Transformation of ligation products into competent *E. coli* cells

Please refer to the study of Yong Zhao [17] for the specific protocol.

#### Screening and identification of recombinant *E. coli* plasmids

The PCR identification system used the universal primers for the vector pCAMBIA1305.2 (Supplementary Table 7). the PCR mix is given (Supplementary Table 8). After the reaction was completed, the PCR products were subjected to agarose gel electrophoresis, and *E. coli* with the correct band size was verified by enzyme digestion and sequencing. Plasmids were extracted from *E. coli* that were confirmed correct by sequencing for transformation of *Agrobacterium tumefaciens*.

### Transformation of *Agrobacterium tumefaciens* with recombinant plasmid

#### Screening and identification of recombinant *Agrobacterium tumefaciens*

The PCR identification system used the universal primers for the vector pCAMBIA1305.2, with the primer sequences and reaction system shown in Supplementary Tables 7 and 8. After the reaction was completed, the PCR products were subjected to agarose gel electrophoresis, and the *Agrobacterium tumefaciens* with the correct band size was stored in the preservation solution for subsequent experiments.

#### Planting, cultivation and transplanting of *A. thaliana*

Under sterile conditions, an appropriate number of full seeds of Arabidopsis (Col-0, *atecp63*, *atddx31-1*, *atddx31-2*, *ata/hd1*) were transferred to sterile centrifuge tubes. Refer to the study of Yong Zhao for the steps of aseptic planting, culture conditions, later transplanting, and cultivation of *A. thaliana* [9].

#### Genetic transformation of *A. thaliana* through floral dipping

*Agrobacterium tumefaciens* containing the *RcECP63*, *RcDDX31*, and *RcA/HD1* genes obtained above was cultured, and the wild-type *A. thaliana* and the *A. thaliana* that was mutant in the three genes were genetically transformed through floral dipping.

#### Screening of transgenic *A. thaliana*

The seeds of T_0_ generation *A. thaliana* harvested after genetic transformation were dried at room temperature for later use. An appropriate number of seeds were inoculated into Murashige and Skoog selection medium (50 mg/mL hygromycin). *A. thaliana* was planted in the steps mentioned before. After 25-28 d of culture, *A. thaliana* seedlings with obvious rooting and good growth were transplanted. When the plants grew to the eight-leaf stage during cultivation, the young leaves were collected for PCR identification. The seeds of the successfully identified plants were harvested for breeding. The homozygous plants were screened according to Mendel's law of heredity, and the T_3_ generation *A. thaliana* plants were selected for phenotypic validation, quantitative analysis, and measurement of physiological indicators.

#### Identification of drought-resistant overexpression and complementary-expression *A. thaliana* plants at the molecular level

PCR identification of drought-resistant overexpression and complementary-expression *A. thaliana* plants: The young leaves (2 mm^2^) of drought-resistant heterologous-overexpression, and complementary-expression *A. thaliana* plants were placed in a sterile Eppendorf tube, identified using the M5 HiPer plus *Taq* HiFi PCR mix, added to 20 μL lysis solution, ground well with a sterile pipette, and boiled for 5 min. After the sample was cooled to room temperature, it was added to 20 μL chloroform. These were mixed by pipetting for 10 s, then centrifuged at 12,000 rpm for 2 min. Two microliters of supernatant was used as the PCR validation template. PCR identification was performed using the universal primers for the vector pCAMBIA1305.2. The primer sequences and reaction system were the same as those in Supplementary Tables 7 and 8. The reaction conditions were 95 °C for 3 min; 36 cycles of 94 °C for 25 s, T_m_ for 25 s, and 72 °C for 1 min 30 s to 2 min 30 s; and lastly 72 °C for 5 min. After the reaction ended, the PCR products were detected by 1% agarose gel electrophoresis to identify the positive overexpression plants *ECP63-OE*, *DDX31-OE*, and *A/HD1-OE* and the positive complementary-expression plants *ECP63-GR*. *DDX31-GR1*, *DDX31-GR2*, and *A/HD1GR*.

RT-qPCR identification of drought-resistant overexpression and complementary-expression A. thaliana plants: The RNA levels of the overexpression A. thaliana plants (*ECP63-OE*, *DDX31-OE*, and *A/HD1-OE*) and the complementary-expression A. thaliana plants (*ECP63-GR*, *DDX31-GR1*, *DDX31-GR2*, and *A/HD1-GR*) under drought stress were identified by analyzing the gene expression variation patterns of both sets. The RNA of the overexpression transgenic plants and complementary- expression transgenic plants in the control and treatment groups were extracted and reverse-transcribed to obtain cDNAs. They were submitted to qPCR with AtActin as the internal reference gene. The primer sequences of AtActin, *RcECP63*, *RcDDX31*, and *RcA/HD1* are shown in Supplementary Table 9.

RT-qPCR identification of resistant overexpression and complementary-expression *A. thaliana* plants: Different concentrations of PEG and mannitol were used to pretreat the four types (wild-type, mutant, overexpression, and complementary expression) of *A. thaliana*. To compare the growth status of *A. thaliana*, the plants were grown in mannitol medium containing 0 mmol/L, 100 mmol/L, 200 mmol/L, and 300 mmol/L mannitol.

Equivalent numbers of homozygous seeds of wild-type, mutant, overexpression and complementary-expression *A. thaliana* were selected and dibbled in the solid MS medium containing 0 mmol/L, 100 mmol, 200 mmol/L, and 300 mmol/L mannitol after sterilization with 75% alcohol under sterile conditions. After vernalization at 4 °C for 3 d, the samples were cultured in a thermostatic incubator. After germination, the germination rates of different types of seeds in the culture medium containing different mannitol concentrations were recorded. After 14 days of culture, the seedlings were photographed and recorded. The culture conditions were a 16-h/8-h light–dark cycle and a 23/20 °C day/night cycle. After the completion of the culture, we compared the root length and leaf growth of each group of *A. thaliana* plants and analyzed the root length and the number of lateral roots of the plants expressing *RcECP63*, *RcDDX31*, and *RcA/HD1* under drought stress treatments with different mannitol concentrations.

#### Measurement of the physiological indicators of four types of *A. thaliana* under the same treatment conditions

The selected *A. thaliana* seeds were sterilized and dibbled in the solid MS medium. After vernalization at 4 °C for 3 d, they were transferred to a thermostatic incubator for culture. Fourteen days later, wild-type, deletion-mutant, overexpression, and complementary-expression *A. thaliana* plants with consistent growth status were transplanted to a medium composed of sterilized nutrient soil:vermiculite at a 3:1 ratio. When the plants reached the eight-leaf stage, they were treated with 10% PEG 6000 (drought stress) for 24 h, 48 h, or 72 h, with water-treated plants as controls, to investigate the changes in four physiological indicators (MDA content, proline content, hydroxyl radical scavenging ability, and T-AOC) of transgenic *A. thaliana* plants under drought stress.

## Discussion and conclusion

### Discussion

The combined proteomic and metabolomic analysis provides more possibilities for exploring and unraveling the complex life activities of organisms. Fan [18] studied the changes in nutritional quality of alfalfa at different growth stages and found that the common metabolic pathways were mainly enriched in pathways related to nutrient metabolism. Durand Thomas et al. [19] performed an combined proteomic and metabolomic analysis on *A. thaliana* seeds and found that the loss of reverse transcriptase stimulates energy metabolism, which in turn affects metabolic pathways, including cell wall biogenesis.

As the end products of gene expression in organisms, metabolites directly or indirectly participate in the metabolic activities of organisms and are most strongly associated with the phenotypes of organisms. Metabolite analyses in Arabidopsis during seed development identified major metabolic abundance transitions associated with successive developmental stages [20]. The detection of 121 differential metabolites by untargeted metabolomics in Japonica and Indica rice seeds revealed a correlation between metabolic phenotypes and the geographical origin of rice seeds [21]. Differential metabolite analysis of wheat seeds identified a total of 74 differential metabolites at developmental stages, and further analyses showed that metabolic pathways for biological processes such as amino acids, carbohydrates, and organic acids are interrelated [22]. Metabolomics provides a reference for better discovering and exploring various complex traits, functions, and features of organisms [23, 24]. Seed germination is essentially related to seed metabolism, and the metabolite content and germination ability of seeds are determined by the potential genetic structures and environmental influences during seed development. Sun [25] found that the vitality and nutritional value of seeds may be related to their chemical compositions. During seed germination, amino acids can play an important role in maintaining osmotic potential.

In this study, the combined proteomic and metabolomic analysis showed that the differentially expressed proteins and differentially expressed metabolites in groups P48_VS_S48 and P60_VS_S60 shared 13 and eight common metabolic pathways, respectively. The combined analysis of the differential proteomics data and comparative metabolomics data of P48_VS_S48 revealed that a total of 13 differential proteins and 13 differential metabolites were annotated to the same metabolic pathways, with the metabolic pathways that shared the same involvement including, Biosynthesis of amino acids, Tyrosine metabolism, 2-Oxocarboxylic acid metabolism, Isoquinoline alkaloid biosynthesis, Carbon metabolism, Phenylalanine metabolism, Phenylalanine, tyrosine and tryptophan biosynthesis, Arginine and proline metabolism, Glyoxylate and dicarboxylate metabolism, Purine metabolism, Tryptophan metabolism, Alanine, aspartate and glutamate metabolism, Citrate cycle, TCA cycle. The combined analysis of the differential proteomics data and comparative metabolomics data of P60_VS_S60 revealed that a total of eight differential proteins and eight differential metabolites were annotated to the same metabolic pathways, and that the metabolic pathways in which the differential proteins and differential metabolites of P60_VS_S60 were jointly involved included Carbon metabolism, Stilbenoid, diarylheptanoid and gingerol biosynthesis, Flavonoid biosynthesis, Citrate cycle, TCA cycle, Biosynthesis of amino acids, Pentose phosphate and gingerol biosynthesis. diarylheptanoid and gingerol biosynthesis, Flavonoid biosynthesis, Citrate cycle, TCA cycle, Biosynthesis of amino acids, Pentose phosphate pathway, Tryptophan metabolism, Glyoxylate and dicarboxylate metabolism.

The physiological significance of the identified common metabolic biological processes, such as the phenylpropanoid biosynthesis, is mainly manifested in their close relationships with physiological activities such as changes in the enzyme system, biosynthesis of intermediate products and their further transformation products, and cell differentiation during plant development. This finding is similar to the findings of previous studies.

In the process of responding to abiotic stress, plants produce drought-induced proteins (LEA, dead box, AK) to adapt to the changing environment. LEA and dead box respond to abiotic stress. Late embryogenesis abundant proteins (LEAs) are a class of proteins that accumulate in large quantities in seeds during the late stages of plant embryogenesis and are an important class of proteins involved in the resistance of organisms to drought stress [26]. In the 1980s, Dure et al [27] first reported the presence of LEA genes in developing seeds of cotton. It was found that the high level of LEA protein expression was closely related to the improvement of drought, salt or low temperature tolerance in plants. The isolation of plant LEA genes with higher stress tolerance is of great significance for research in plant adversity stress [28]. Dead box ATP-dependent RNA helicase is a family of Dead-box ATP-dependent RNA deconjugating enzymes, and the DEAD-box (DDX) family is a family of RNA deconjugating enzymes that are of critical to animal embryo development and has received much attention in recent years. In plants, 32 genes of the DDX deconjugulase family were identified for the first time in Arabidopsis thaliana [29], followed by several other RNA deconjugulases regulating plant growth and stress [30–32], which demonstrated that the DDX gene family is involved not only in plant growth and development, but also abiotic stresses such as salt, osmotic, drought stress, cold and phytohormones, including gametophyte and embryo development in Arabidopsis thaliana [33, 34]. Aspartate kinase (AK) is a rate-limiting enzyme that restricts the flow rate of carbon and nitrogen sources, and AK is a large class of protein kinases, which has a wide range of applications in fields such as pharmaceutical fermentation, and is one of the key determinants of the ability to obtain high amino acid yields [35].

Although AK expression is greatly upregulated under stress, there is no relevant research on its role in plant stress resistance. In addition, many protein kinases are highly expressed in response to abiotic stress. In the phenotypic validation of the three differentially expressed genes selected in this study, we found that the common metabolic pathways of the screened differentially expressed proteins and differentially expressed metabolites all play an important role in plant response to biotic stress.

The ideal and commonly used drought stress agents include PEG and mannitol. PEG can be used to simulate soil drought stress [36]. In this study, mannitol concentrations of 0 mmol/L, 100 mmol/L, 200 mmol/L, and 300 mmol/L were selected to distinguish the phenotypes of different types of *A. thaliana* plants treated with different concentrations of PEG and different concentrations of mannitol.

## Conclusion

In this study, three differentially up-regulated genes, *RcECP 63*, *RcDDX 31* and *RcA/HD1*, were finally selected and analysed for gene function by RT-qPCR analysis of the histological data and selected genes encoding differential proteins. The silencing treatment of *castor* leaves revealed that castor leaves were more intolerant to drought stress after silencing the three genes, and it was hypothesised that the three selected genes play an important role in the response to drought stress during the germination period of castor. Mannitol tolerance analysis of four types of Arabidopsis thaliana, namely, mutant, back-expressed, wild-type and overexpressed, revealed that the three selected genes were important in the response to drought stress. Laying the foundation for the study of drought tolerance mechanism in *castor*.

### Supplementary Information

**Supplementary Material 1.** 

**Supplementary Material 2.** 

## Data Availability

Data and materialshas are all availability.
