# Supplementary material for: Screening and functional verification of drought resistance-related genes in castor bean seeds
Source: BMC Plant Biol. 2024 Jun 3;24:493. doi: 10.1186/s12870-024-04997-7 (PMC11145773; doi:10.1186/s12870-024-04997-7)
Supplement: Supplementary file 1 — Supplementary Material 1. [file 12870_2024_4997_MOESM1_ESM.pdf]

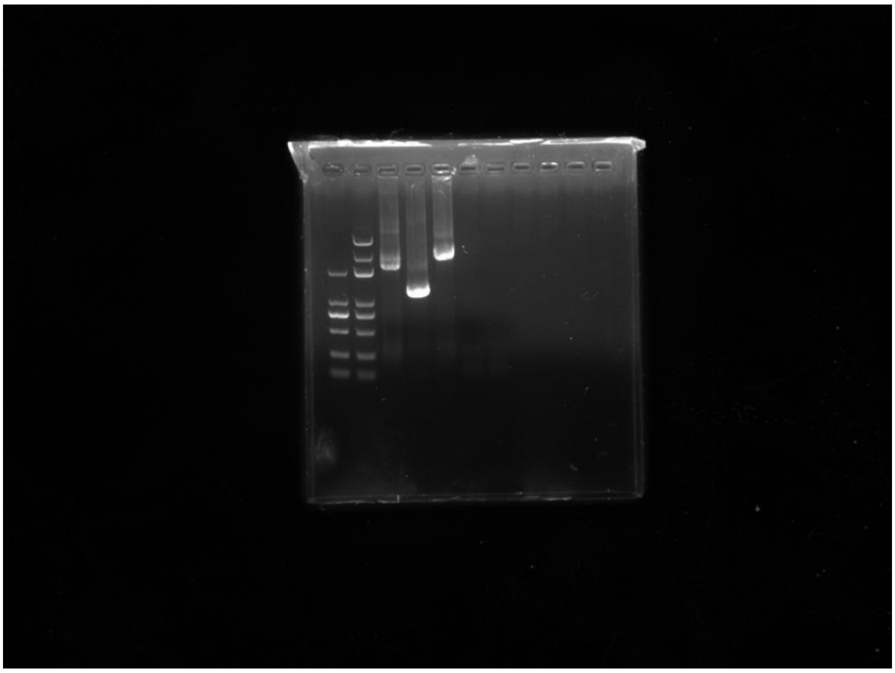

Figure 15 PCR amplification results of *RcDDX31*, *RcECP63*, and *RcA/HD1*
Note: M: DL 5000 Marker; 1: *RcDDX31*; 2: *RcECP63*; 3: *RcA/HD1*

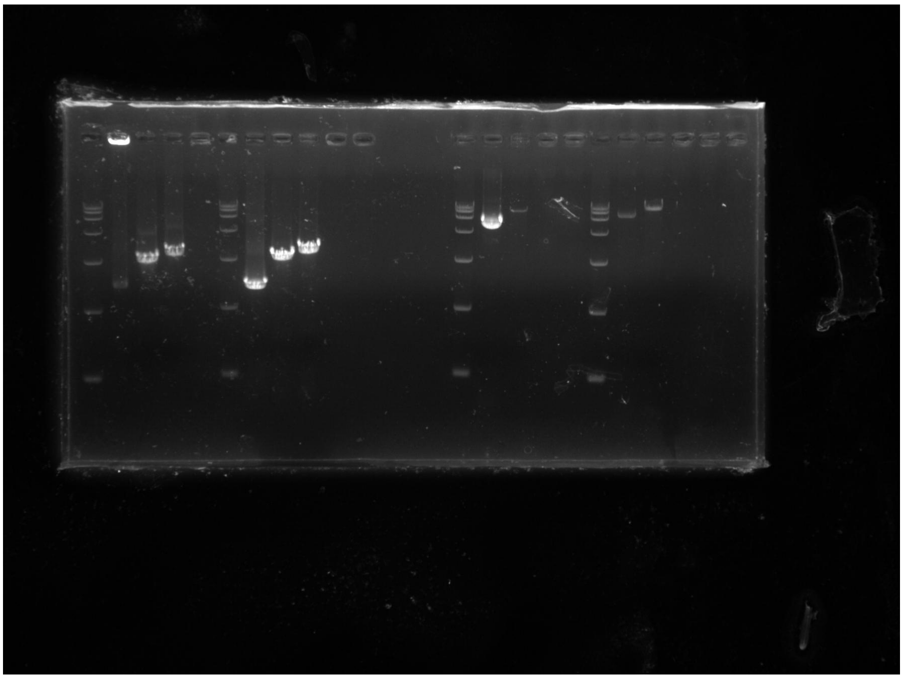

Figure 16 Results of single-enzyme digestion of the heterologous expression vector pCAMBIA1305.2
Note: M: DL 15000 Marker; 1: plasmid control; 2: enzyme digestion product
（the third one is right）

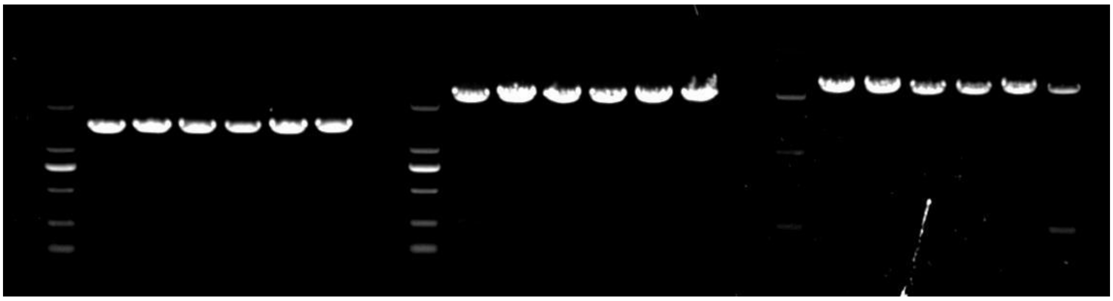

Figure 17 PCR results of *E. coli* recombinant plasmids
Note: M1 and M2: DL 2000 Marker; 1-6: *RcECP63*; 7-12: *RcDDX31*; 13-18: *RcA/HD1*

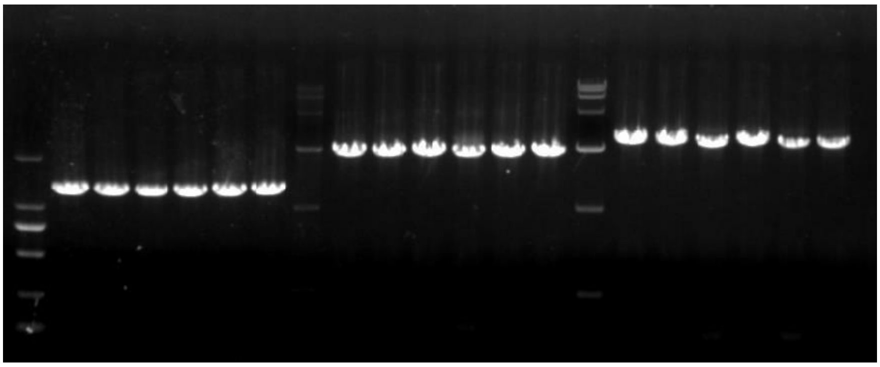

Figure 18 PCR results of *Agrobacterium tumefaciens* recombinant plasmids
Note: M1: DL 2000 Marker; M2, M3: DL 15,000 Marker; 1-6: *RcECP63*; 7-12: *RcDDX31*; 13-18: *RcA/HD1*

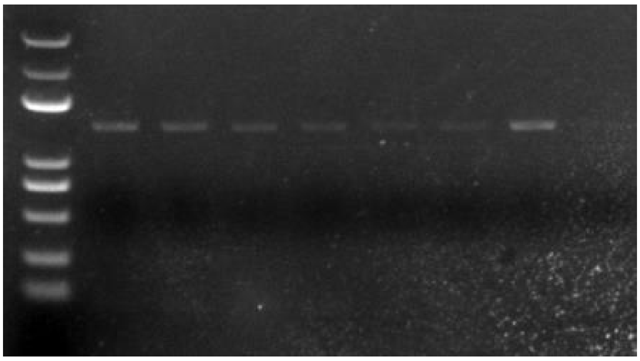

Figure 19(1) Results of PCR identification of the resistant *Arabidopsis thaliana* plants with overexpression and complementary expression of *RcECP63*
Note: M1 and M2: DL 5000 Marker; 1-8: PCR identification results of the resistant complementary-expression *A. thaliana* plant ECP63-GR

9-15: PCR identification results of the resistant overexpression *A. thaliana* plant ECP63-OE

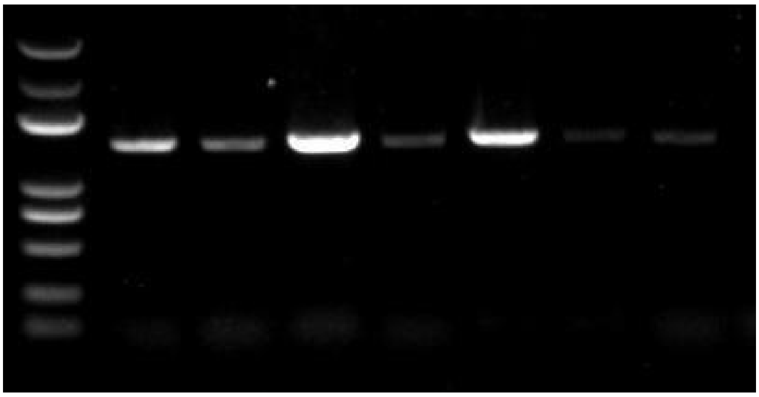

Figure 19(2) Results of PCR identification of the resistant *Arabidopsis thaliana* plants with overexpression and complementary expression of *RcECP63*

Note: M1 and M2: DL 5000 Marker; 1-8: PCR identification results of the resistant complementary-expression *A. thaliana* plant ECP63-GR

9-15: PCR identification results of the resistant overexpression *A. thaliana* plant ECP63-OE

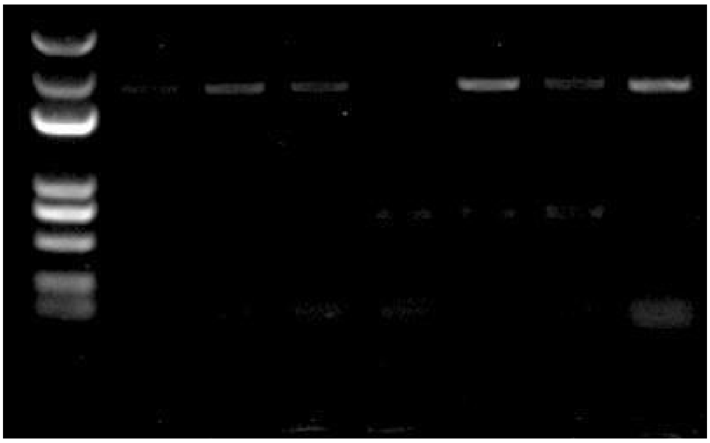

Figure 20(1) Results of PCR identification of the resistant *Arabidopsis thaliana* plants with overexpression and complementary expression of *RcDDX31*

Note: M1, M2, and M3: DL 5000 Marker; 1-7: PCR identification results of the resistant complementary-expression *A. thaliana* plant DDX31-GR1; 8-15: PCR identification results of the resistant complementary-expression *A. thaliana* plant DDX31-GR2; 16-23: PCR identification results of the resistant overexpression *A. thaliana* plant DDX31-OE

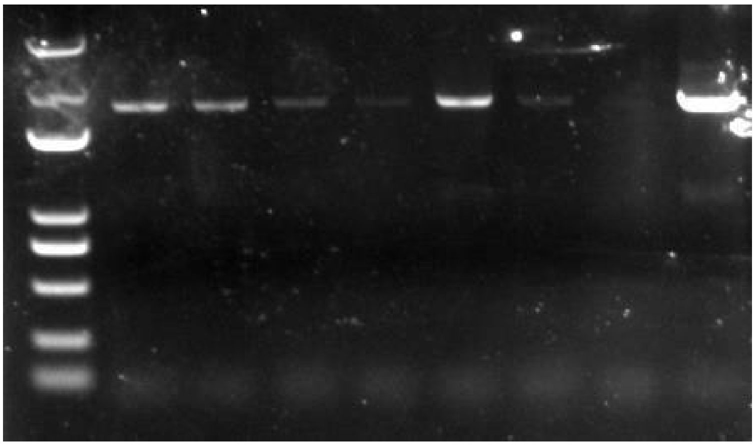

Figure 20(2) Results of PCR identification of the resistant *Arabidopsis thaliana* plants with overexpression and complementary expression of *RcDDX31*

Note: M1, M2, and M3: DL 5000 Marker; 1-7: PCR identification results of the resistant complementary-expression *A. thaliana* plant DDX31-GR1; 8-15: PCR identification results of the resistant complementary-expression *A. thaliana* plant DDX31-GR2; 16-23: PCR identification results of the resistant overexpression *A. thaliana* plant DDX31-OE

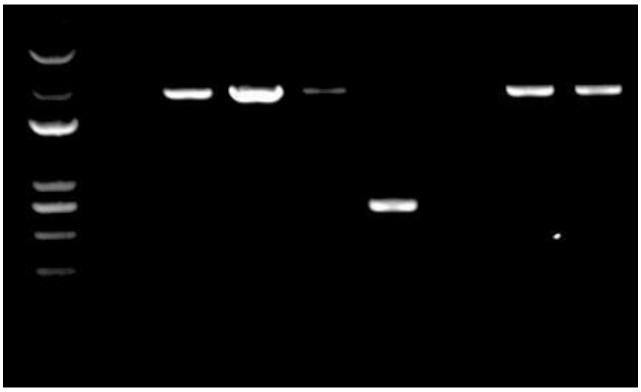

Figure 20(3) Results of PCR identification of the resistant *Arabidopsis thaliana* plants with overexpression and complementary expression of *RcDDX31*

Note: M1, M2, and M3: DL 5000 Marker; 1-7: PCR identification results of the resistant complementary-expression *A. thaliana* plant DDX31-GR1; 8-15: PCR identification results of the resistant complementary-expression *A. thaliana* plant DDX31-GR2; 16-23: PCR identification results of the resistant overexpression *A. thaliana* plant DDX31-OE

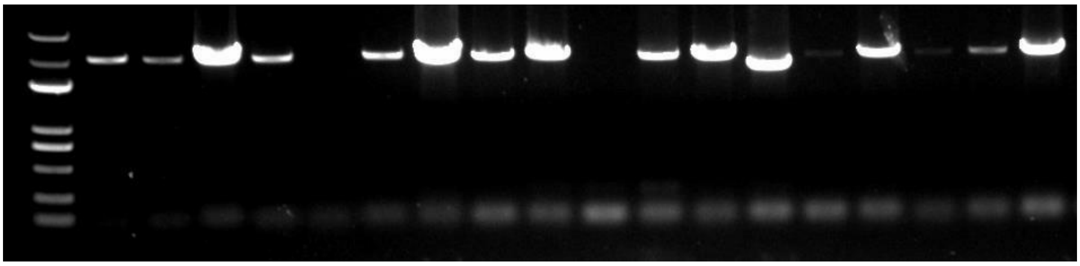

Figure 21(1) Results of PCR identification of the resistant *Arabidopsis thaliana* plants with overexpression and complementary expression of *RcA/HD1*

Note: M1 and M2: DL 5000 Marker; 1-6: PCR identification results of the resistant complementary-expression *A. thaliana* plant A/HD1-GR; 7-13: PCR identification of the resistant overexpression *A. thaliana* plant A/HD1-OE

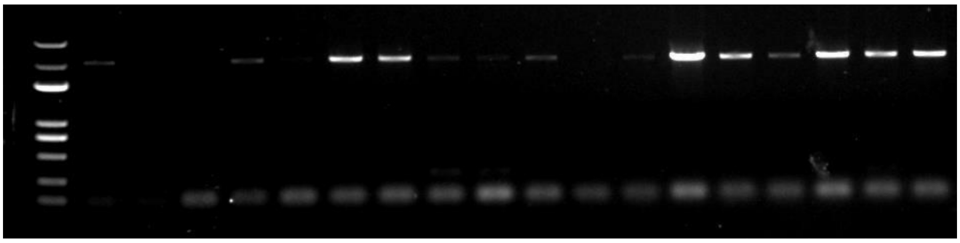

Figure 21(2) Results of PCR identification of the resistant *Arabidopsis thaliana* plants with overexpression and complementary expression of *RcA/HD1*

Note: M1 and M2: DL 5000 Marker; 1-6: PCR identification results of the resistant complementary-expression *A. thaliana* plant A/HD1-GR; 7-13: PCR identification of the resistant overexpression *A. thaliana* plant A/HD1-OE

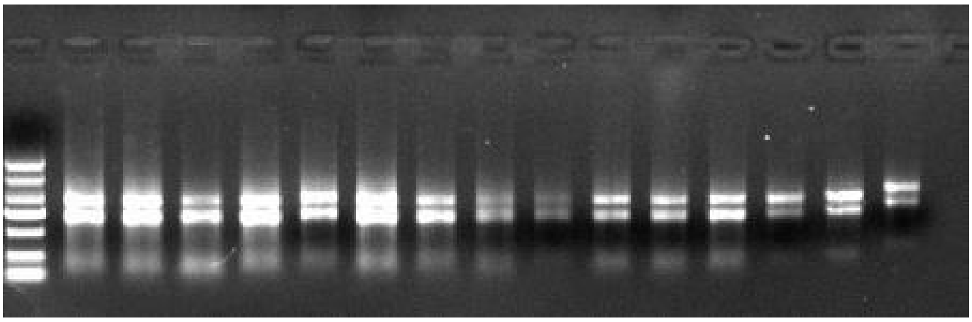

Figure 23 RNA extraction Results of positive complementary-expression *Arabidopsis thaliana* plants

Note: M: DL 2000 Marker; 1-4 represent the RNA extraction results of the compleme ntary-expression plant ECP63-GR; 5-8 represent the RNA extraction results of the complementary-expression plant DDX31-GR1; 9-12 represent the RNA extraction results of the complementary-expression plant DDX31-GR2; 13-15 represents the RNA extraction results of the complementary-expression plant A/HD1-GR
