# Supplementary material for: Screening and functional verification of drought resistance-related genes in castor bean seeds
Source: BMC Plant Biol. 2024 Jun 3;24:493. doi: 10.1186/s12870-024-04997-7 (PMC11145773; doi:10.1186/s12870-024-04997-7)

Table 1 qPCR primer sequences for genes encoding differentially expressed proteins


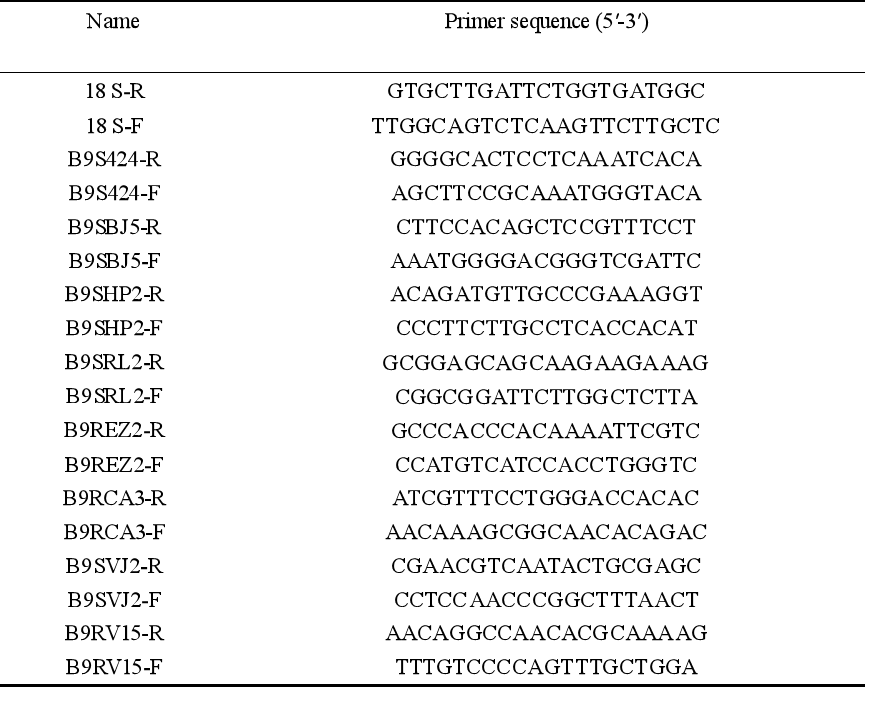


Table 2 RT-qPCR systems for genes encoding differentially expressed proteins


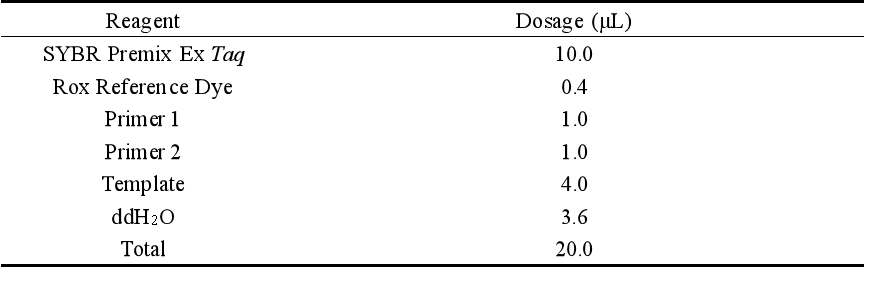


Table 3 PCR primer sequences for the target genes


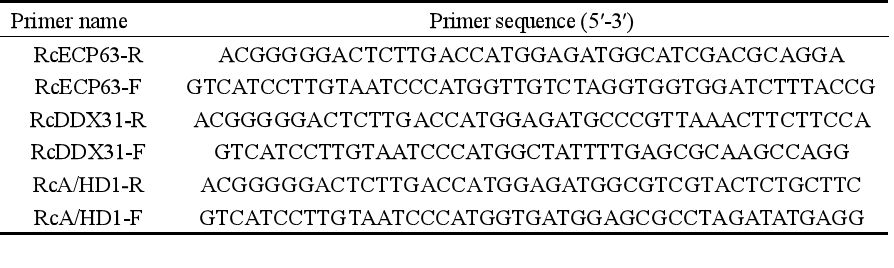


Table 4 PCR system for the target genes


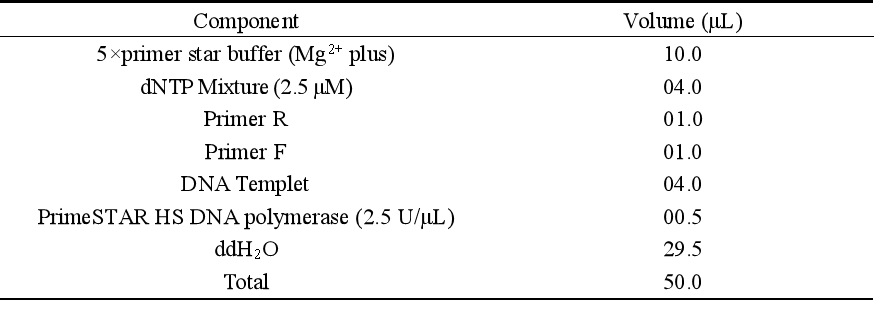


Table 5 Single-enzyme digestion system of the heterologous expression vector *Nco*I


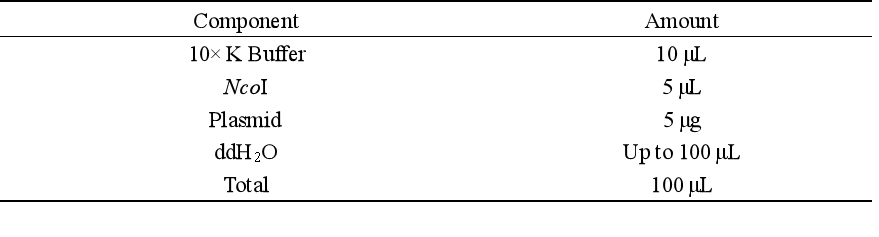


Table 6 Ligation system between the target genes and the heterologous expression vector


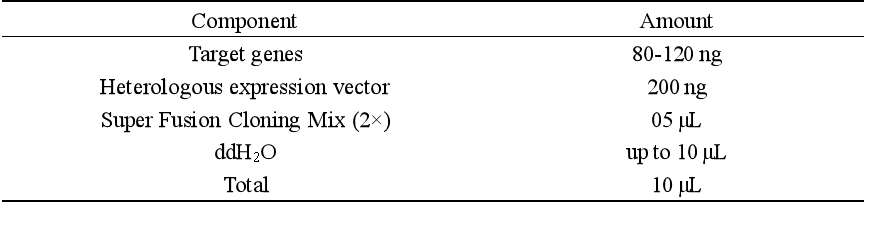


Table 7 Universal primers for the vector pCAMBIA1305.2

**
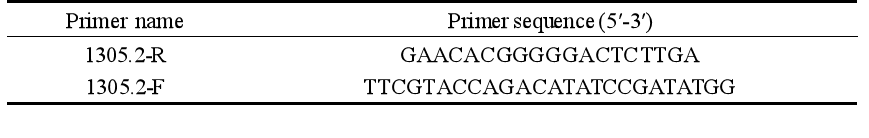
**

Table 8 PCR system for the recombinant plasmids

**
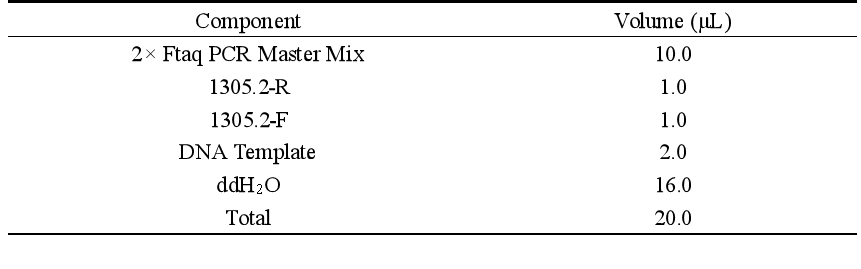
**

Table 9 qPCR primer sequences


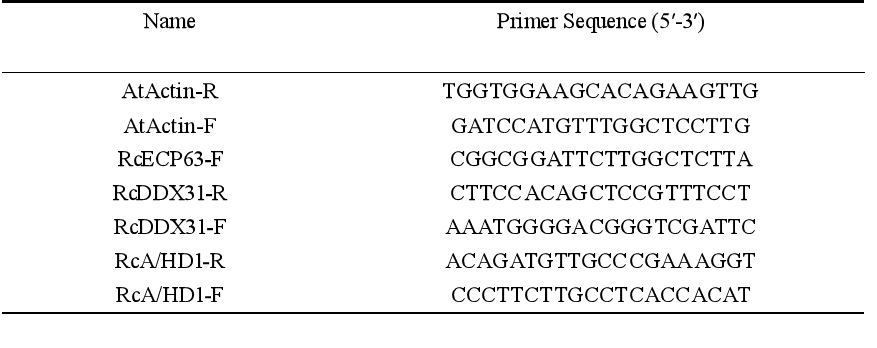

Supplement: Supplementary file 2 — Supplementary Material 2. [file 12870_2024_4997_MOESM2_ESM.docx]
